# Fluorescent Diarylethenes With Polar Groups: Synthesis, Spectra, and Optical Microscopy Applications

**DOI:** 10.1002/chem.202503087

**Published:** 2025-12-24

**Authors:** Kakishi Uno, Sven Nagorny, Ayse Aktalay, Dojin Kim, Mariano L. Bossi, Vladimir N. Belov, Stefan W. Hell

**Affiliations:** ^1^ Department of NanoBiophotonics Max Planck Institute For Multidisciplinary Sciences (MPINAT) Göttingen Germany; ^2^ Department of Optical Nanoscopy Max Planck Institute For Medical Research Heidelberg Germany

**Keywords:** chromophores, diarylethenes, fluorescence, photochemistry, photochromism

## Abstract

The use of photoactivatable fluorescent diarylethenes (fDAEs) in biology‐related light microscopy has been restricted by the lack of probes having freely variable structural and spectral properties. Facing these challenges, we synthesized “turn‐on” fDAEs with four hydroxyl groups and a single functionality in the core structure (HO‐fDAEs). HO‐fDAEs emit green light; they were used for the preparation of cell‐permeant mitochondrion‐selective probes and bioconjugation with proteins (via a single COOH group). HO‐fDAEs were combined in bio‐imaging with a thiophene‐substituted fDAE (Th‐fDAE) possessing red‐shifted absorption and emission bands of the open‐ and closed‐ring isomers. Th‐fDAE undergoes full on‐switching with 405 nm and excitation with green light (< 560 nm). The large Stokes shifts of HO‐and Th‐fDAE (80 and 110 nm, respectively) and different excitation wavelengths permitted color separation in optical microscopy. Photoactivation of HO‐fDAEs and transient on‐off switching (blinking) of Th‐fDAE made it possible to acquire two‐color images of cellular structures with optical superresolution.

## Graphical abstract

Blinking green and flashing red: photo‐switchable and photo‐activatable probes emitting green and red light were prepared and applied as bioconjugates in super‐resolution optical microscopy.

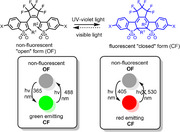

## 1 Introduction

In 1867, M. Fritzsche reported that an orange‐colored tetracene solution bleached when exposed to sunlight but recolored during the night [1]. In a more general sense, the reversible color change was called photochromism: the ability of a compound to reversibly switch between two different states upon irradiation with light [2]. In most cases, new chemical bonds are formed and broken intramolecularly during this transition, and the two states represent isomeric structures. If both isomers are thermally stable, illumination with light of two distinct wavelengths is required for the interconversion [3].

Diarylethenes (DAEs), classified as P‐type photochromic molecules, have now become a versatile platform of constructing optical/functional materials, because their open form (OF) and closed form (CF) are thermally stable and the absorption bands of both forms are well separated [4, 5, 6, 7, 8]. They undergo fatigue‐resistant and efficient photoswitching reactions. Merging the photochromic DAEs and fluorescent dyes into a single entity deserved particular attention over the past decades [5, 9, 10, 11, 12]. The resulting photo‐responsive fluorescent dyes are expected to become useful molecular switches applicable in life [13, 14, 15, 16, 17, 18, 19] and materials science [20, 21, 22, 23, 24, 25, 26]. In particular, superresolution microscopy (SRM), which enables to achieve optical resolution down to nanometer scales, relies on light‐induced transitions between nonfluorescent (*off*) and fluorescent (*on*) molecular states [27, 28, 29, 30, 31]. When the photochromic and fluorescent parts are connected via linkers, the emission can be modulated via Förster resonance energy transfer (FRET) [20, 32, 33] or photoinduced electron transfer (PET) [34], in parallel with the progress of the photochromic reaction. In most cases, however, this molecular design leads to *turn‐off* fluorescence switches (the colored form is nonfluorescent) with low *on/off* or signal‐to‐background (S/N) ratios, and this negatively influences the SRM image.

Recently, *turn‐on* mode fluorescent diarylethenes (fDAEs) bearing two benzothiophene‐1,1‐dioxide residues attached to perfluorocyclopentene ring have been reported [35, 36, 37, 38, 39]. They possess unique properties for SRM: in situ fast generation of the emissive CF exhibiting high brightness and large Stokes shift (a rare combination) [40], complete back‐conversion to the nonfluorescent OF, which leads to high emission contrast [41], and photo‐fatigue resistance (repeated *on/off* photoswitching) [42]. For applications in aqueous media, in particular protein labeling, we developed water‐soluble fDAEs decorated with numerous carboxylic acid residues and demonstrated their applications in optical SRM, such as RESOLFT (reversible saturable optical linear fluorescent transitions) [28, 43, 44, 45] and STORM (stochastic optical reconstruction microscopy) [29, 46, 47]. RESOLFT methods require fast‐switching probes providing multiple on‐off cycles, thus phenylated fDAEs with asymmetric *tetra*‐acid [44, 45] and symmetric *tetra*‐ and *octa*‐acid substitution patterns were developed [41, 43]. In contrast, as STORM requires probes providing a low number of cycles with a high photon turnover per cycle, “slow” fDAEs with electron‐donating 4‐methoxyphenyl group(s), which reduce the quantum yield of the ring‐opening reaction, excelled at this task [41].

Despite the success in the development of fDAEs for protein (e.g., antibodies, nanobodies) labeling and immunostaining, the major limitations of current fDAEs restricting their use in living cells and in (multicolor) bio‐imaging, including SRM are 1) lack of cell‐permeant probes having functional groups and uncharged polar residues and 2) the absence of fDAE pairs and imaging schemes providing independent detection of two markers with an option of optical superresolution [41, 43, 44, 45].

**SCHEME 1:**
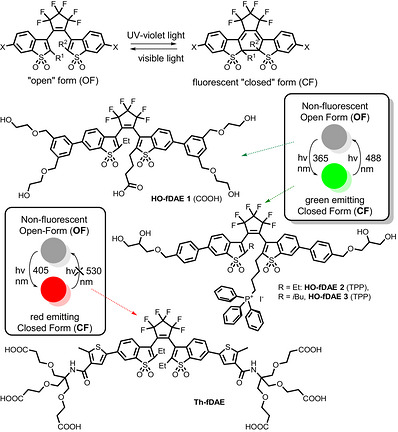
*Top*: general structure and isomerization reaction of fluorescent turn‐on mode diarylethenes (fDAEs). *Center*: **HO‐fDAEs 1**, **2,** and **3** emitting green. *Bottom*: **Th‐fDAE** emitting red light.

**SCHEME 2:**
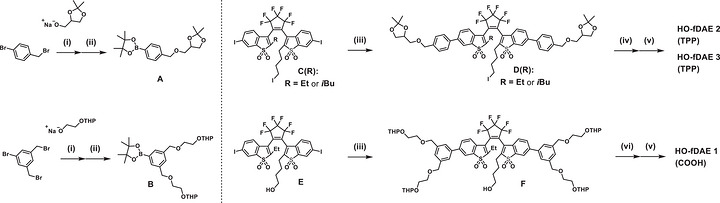
Synthesis of green‐emitting **HO‐fDAEs 1**, **2,** and **3**. Reagents and conditions: (i) THF, 0°C to r. t., 12 h. (ii) (BPin)_2_, AcOK, Pd(dppf)Cl_2_, 1,4‐dioxane, 90°C, overnight. (iii) **A** or **B**, Pd(dba)_2_, Sphos, K_2_CO_3_, aqueous THF, reflux, 30 min. (iv) PPh_3,_ ACN, reflux, 2 h. (v) PPTS, MeOH, reflux 3 h. (vi) TEMPO/BAIB, ACN/H_2_O (1/1, v/v), r. t., 30 min.

Here we introduce green‐emitting HO‐fDAEs bearing a single functional group (carboxyl) or triphenylphosphonium (TPP) tag at one of the central carbon atoms and four hydroxyl residues attached via ether‐containing linkers to phenyl substituents (Schemes 1 and 2). This provides functional dyes with sufficient water solubility and cell permeability, allowing for live‐cell labeling and imaging. The photochromic core undergoes a ring‐closure reaction with 355–365 nm light. The closed‐ring isomers are highly fluorescent under irradiation with blue to green light and have large Stokes shifts.

As a “second‐color dye,” we applied **Th‐fDAE** (Scheme 1) possessing red‐shifted absorption and emission bands of the open‐ and closed‐ring isomer (compared with HO‐fDAEs) [46]. Th‐fDAEs undergo full on‐switching with 405 nm and excitation with green light (< 560 nm). The presence of thienyl groups enhances the Urbach tail effect [46, 47], which allows single‐molecule *on*/*off* “blinking” with only one irradiation source (below 560 nm). Taking advantage of the spectral features, photoactivation, and excitation wavelengths, we prepared conjugates with antibodies and applied them as photoswitchable markers in confocal and coordinate‐based stochastic superresolution microscopy.

## 2 Results and Discussion

### 2.1 Dye Design and Synthesis

Two boronic esters (**A** and **B**)decorated with tetrahydro‐2*H*‐pyran (THP) or 2,2‐dimethyl‐1,3‐dioxolane (DDO) residues as protecting groups for alcohols (OHs) were synthesized in two steps (Scheme 2). First, the in‐situ generated sodium alkoxides of (2,2‐dimethyl‐1,3‐dioxolan‐4‐yl)methanol and 2‐((tetrahydro‐2*H*‐pyran‐2‐yl)oxy)ethan‐1‐ol were introduced into reactions with 1‐bromo‐4‐(bromomethyl)benzene and 1‐bromo‐3,5‐bis(bromomethyl)benzene, respectively. Then, the aryl bromo residues were converted to boronic esters via Miyaura‐borylation reaction to give building blocks **A** and **B**. Compounds **HO‐fDAE 2** (TPP) and **HO‐fDAE 3** (TPP) bearing positively charged triphenylphosphonium residue (Scheme 1) for targeting mitochondria were prepared from iodides **C(Et)** and **C(*i*Bu)** that possess a single ω‐iodobutane chain as a reactive site for triphenylphosphine. The detailed preparation procedures for the syntheses of DAE units **C(Et)** and **C(*i*Bu)** are described in Supp. Inf. Suzuki‐Miyaura coupling reactions using DAEs **C(Et)**/**C(*i*Bu)** and boronic ester **A** were performed to give intermediates **D(Et)**/**D(*i*Bu)** in good yields. Triphenylphosphine (PPh_3_) reacted with primary alkyl iodide residues in **D(Et)**/**D(*i*Bu)** at reflux in acetonitrile to form alkyl triphenylphosphonium tags. Lastly, dioxolane groups were cleaved off under mild conditions to provide **HO‐fDAE 2** (TPP) and **HO‐fDAE 3** (TPP) as final products.

Carboxylic acid **HO‐fDAE 1** was synthesized by Suzuki‐Miyaura coupling reaction of precursors **E** and **B,** oxidation of alcohol to carboxylic acid, and removal of THP protection. Overall, this approach creates asymmetric DAE structures with numerous hydroxyl groups as polar residues and a single pre‐installed reactive group, which enables bioconjugation and expands its scope. This reactive group originates from an alcohol or alkyl iodide, as versatile functional groups that may easily be converted to carboxylate, azide, amino, maleimido, and other functionalities.

### 2.2 Photo‐physical Characterization

**FIGURE 1:**
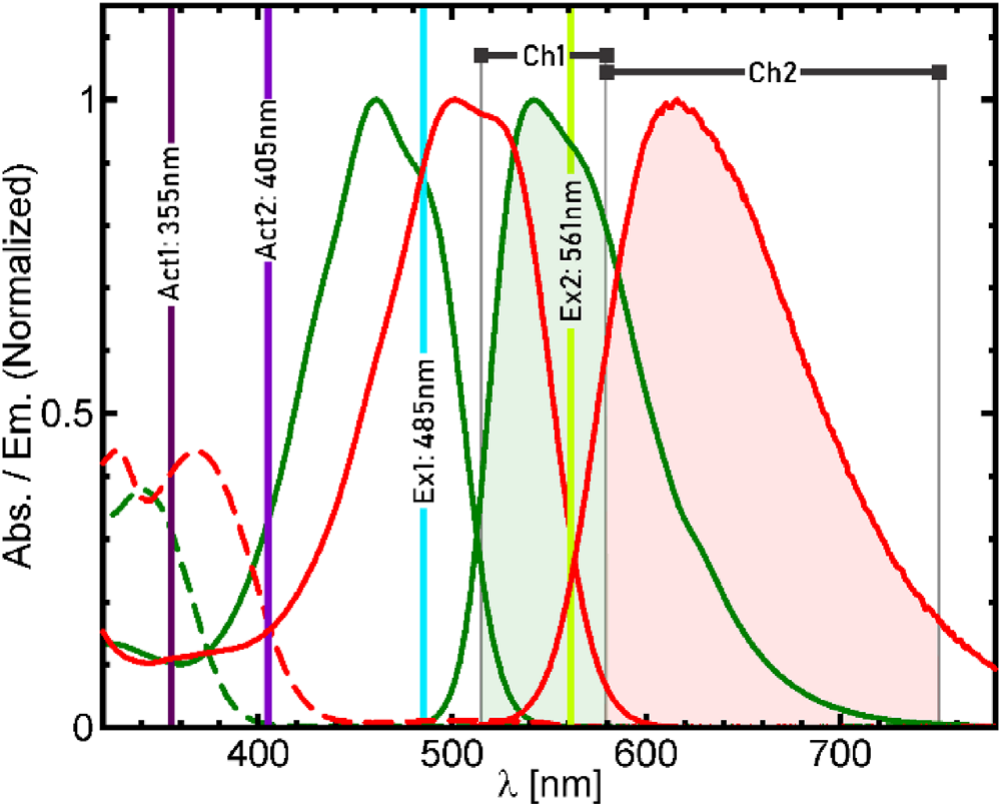
Absorption spectra of the “open” (dashed lines) and “closed” forms (CFs, solid lines) of **HO‐fDAEs 1** (green) and **Th‐fDAE** (red), with structures shown in Scheme 1. Solvent: acetonitrile. Irradiation with 355 nm light (Act1) for **HO‐fDAEs 1** and 405 nm light (Act2) for **Th‐fDAE** provides fluorescent CFs. The normalized emission bands (with large Stokes shifts) are shown for compounds **HO‐fDAEs 1** and **Th‐fDAE** (in their CFs) as green and red lines, respectively. Excitation wavelengths (Ex1, Ex2) and detection windows (Ch1, Ch2) used for two‐color imaging (Figure 4) are shown.

The photophysical properties of fDAEs (see Table 1) were studied in acetonitrile and buffered aqueous solutions (phosphate buffer, 100 mM, pH = 7). Irradiation with UV light (365 nm) caused the cyclization of **HO‐fDAEs 1**, **2,** and **3**, while irradiation with violet light (405 nm) resulted in ring‐closure of **Th‐fDAE** [46]. Complete conversions were observed in the photostationary state for all compounds. The cycloreversion reactions were induced with light in the range of 470–488 nm (blue to blue‐green) for **HO‐fDAEs 1**, **2,** and 530 nm (green) for **Th‐fDAE**. The absorption spectra of OF and CF and the normalized fluorescence spectra of CF are shown in Figure 1. All **HO‐fDAEs** have a similar chromophore whose absorption and emission bands are blue‐shifted compared with that of **Th‐fDAE** [46].

**TABLE 1:** Photophysical properties of fDAEs in acetonitrile and aqueous buffered solutions (pH = 7, phosphate buffer 100 mM)

	*λ* ^max^_abs_ [nm] / *ε*×10^−3^ [M^−1^cm^−1^]^[^ ^a^ ^]^		*λ* ^max^_em_ [nm]	*Φ*_fl_^[^ ^b^ ^]^ CF		*Φ*_isom_ / MeCN		*Φ*_isom_ / aq. buffer		N_1/2_ (solv.)^[^ ^e^ ^]^
	OF^[^ ^a^ ^]^	CF	CF^[^ ^c^ ^]^	MeCN	aq. buffer	OF→CF^[^ ^c^ ^]^	CF→OF^[^ ^d^ ^]^	OF→CF^[^ ^c^ ^]^	CF→OF^[^ ^d^ ^]^	
**HO‐fDAE 1**	345/14.1	460/37.2	532	0.38	0.12	0.30	1.5×10^−3^	0.20	1.4×10^−3^	640 (MeCN) 23 (H_2_O)
**HO‐fDAE 2**	340/20.2	460/55.0	545	0.38	n.d.	0.23	5.3×10^−3^	n.d.^[^ ^f^ ^]^	n.d.^[^ ^f^ ^]^	2864 (MeCN) n.d. (H_2_O)^[^ ^f^ ^]^
**HO‐fDAE 3**	340/14.1	461/37.2	546	0.64	0.38	0.21	2.4×10^−3^	0.15	1.5×10^−3^	2675 (MeCN) 60 (H_2_O)
**Th‐fDAE** ^[^ ^g^ ^]^	369/19.3	504/43.7	608	0.65	0.34	9.2×10^−4^	3.3×10^−6^	2.9×10^−4^	3.8×10^−6^	irreversible

^[a]^ lowest energy absorption peak

^[b]^ fluorescence quantum yield

^[c]^ measured at 365 nm (HO‐fDAEs) or 405 nm (Th‐fDAE)

^[d]^ measured at 470 (HO‐fDAEs) or 530 nm (Th‐fDAE; see *Supporting Information* for details)

^[e]^ photo‐fatigue resistance: number of switching cycles before 50% loss in the fluorescence signal is observed

^[f]^ values not determined in water due to aggregation of the CF

^[g]^ ref. [46].

Fluorophores with large Stokes shifts and high values of emission efficiencies are rare. For the closed‐ring isomers of fDAEs having large Stokes shifts (80‐110 nm), the fluorescence quantum yields were found to be relatively high [37, 41, 43, 46, 47, 48]. The CFs of **HO‐fDAEs** and **Th‐fDAE** may be readily excited with commercial 485 nm and 561 nm lasers, respectively. Thus, **HO‐fDAEs** and **Th‐fDAE** represent a set of combinable photoswitchable and photoactivatable fluorescent dyes with large Stokes shifts for two‐color applications based on selective excitation and emission discrimination. Their extinction coefficients are 37.000–55.000 M^−1^cm^−1^, so that the brightness (product of the absorptivity at the excitation wavelength and the emission efficiency) is in the range of coumarins, which are considered to be benchmark dyes with large Stokes shifts.

The isobutyl (*i*Bu) group was introduced to the central carbon atom (C‐2') of **HO‐fDAE 3** in order to increase the cycloreversion quantum yield [39, 40, 42, 48]. The values of cycloreversion quantum yields for compound **HO‐fDAE 3** in both solvents were two orders of magnitude lower than cyclization efficiencies but measurable (unlike for compound **HO‐fDAE 2,** which aggregated in aqueous buffer). In fact, for each compound in Table 1, the cycloreversion quantum yields are much lower than the cyclization quantum yields. This ensures a complete conversion in the PSS and a reasonable number of photons emitted per ON‐cycle. **HO‐fDAEs** show high fatigue resistance in acetonitrile. The values of *N*_1/2_ are defined as the numbers of full ON‐OFF (or OFF‐ON) switching cycles before 50% bleaching occurs. We observed up to 2800 cycles in acetonitrile (for **HO‐fDAE 2)**, but only 23 or 60 cycles in aqueous solutions for **HO‐fDAEs 1** and **HO‐fDAE 3**, respectively. The cycloreversion quantum yield of **Th‐fDAE** is particularly low (3*10^−6^), which is, in fact, advantageous for applications requiring single‐molecule detection [49]. When *Φ*_CF→OF_ ≤ 10^−6^, the photo‐switching becomes practically irreversible, because bleaching outperforms ring opening. Thus, in the second cycle, compound **Th‐fDAE** in aqueous solutions only produced ca. 35% of the CF formed in the first cycle.

**FIGURE 2:**
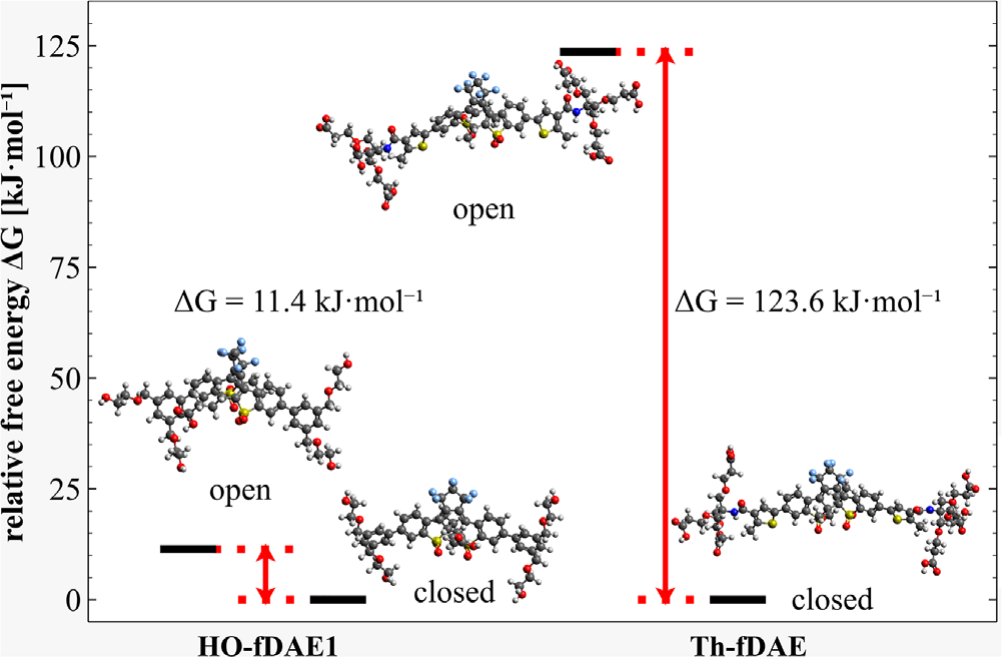
Relative free energies (ΔG, kJ·mol^−1^, B3LYP/6‐31G(d,p) + D3BJ, SMD (H_2_O) of the open‐ and closed‐ring isomers of **HO‐fDAE1** and **Th‐fDAE**. Values are given relative to the most stable minimum of each system. Coordinates and full sets of data are given in Supporting Information.

To understand the markedly different photochemical behavior of **Th‐fDAE,** we carried out ground‐state DFT calculations in an aqueous environment (see Supporting Information for details). **HO‐fDAE 1** was chosen as reference. The results (Figure 2) show that the closed form is thermodynamically favored for both systems, but to very different extents: **HO‐fDAE 1** has a modest free‐energy preference for the closed form (ΔG = 11.4 kJ·mol^−1^), whereas **Th‐fDAE** exhibits a very large stabilization of this form (ΔG  =  123.6 kJ mol^−1^). A deep energy minimum of the closed‐ring isomer **Th‐fDAE** in the ground state (S_0_) can be explained by 2 factors. First, the enhanced π‐conjugation with the electron‐deficient core is provided by thienyl units (which are known to lower the quantum yields of the ring‐opening reaction [50, 51]). Second, upon ring‐closure, two ethyl groups in **Th‐fDAE** afford less steric hindrance than ethyl and 3‐carboxypropyl groups in **HO‐fDAE 1**. The latter factor allows benzothiophene units to adopt a geometry that provides better  conjugation in **Th‐fDAE_,_** as compared with **HO‐fDAE 1**.

Analysis of the optimized ground‐state geometries shows only minor differences in the reactive C‐C bond distances between open and closed rings. Although small geometric changes in the S_0_ minima do not by themselves determine the kinetics, the large ΔG value computed for **Th‐fDAE** explains its slow cyclo‐reversion behavior, especially in water, relative to **HO‐fDAE1**.

When the solutions containing only the OF of **Th‐fDAE** were exposed to blue to green light (i.e., 450–530 nm), a photo‐equilibrium containing a relatively large fraction of the CF was reached, though *apparently* only the CF absorbs in this region (*a priori* we could expect that all amount of the CF would have been converted into OF). In fact, the cyclization reaction induced by visible light is due to the so‐called Urbach tail absorption of the OF in the 450–530 nm range [46, 47]. The Urbach tail effect − a photo‐induced cyclization reaction by absorption of weak “hot” bands of the OF − can also be observed for other fDAEs. However, in this and a previous study [46], it was used for inducing “blinking” of individual fluorophores **Th‐fDAE** and imaging by means of single‐molecule switching between “dark” (OF) and “bright” (CF) states.

### 2.3 Mitochondria Probes

**FIGURE 3:**
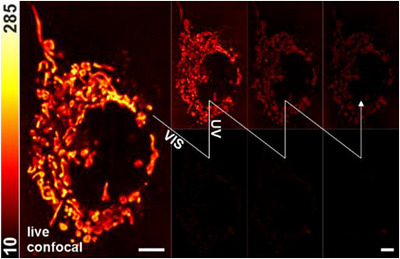
Mitochondria probe based on the fast‐switching fDAEs with four hydroxyl groups and ethyl (**HO‐fDAE 3**). Labeling was performed with the closed‐ring fluorescent isomers. Note the limited number of switching cycles.

We prepared two mitochondria probes based on the fast‐switching fDAEs with four hydroxyl groups and one ethyl (**HO‐fDAE 2**) or isobutyl (**HO‐fDAE 3**) residue attached to C‐2 of the benzothiophene unit. On the opposite inner side of the switching unit (at C‐2´), there was the positively charged 4‐(triphenylphosphonio)butyl substituent, responsible for binding with mitochondria. In confocal microscopy, these probes demonstrated specific staining of mitochondria in live COS7 cells confirmed by colocalization with MitoTracker Deep Red (Figures 3 and S1). Applying probes containing considerable amounts of the closed‐ring (emissive) isomers, we observed the fluorescent mitochondria, and their emission signal could not be further increased by irradiation with UV light. Under these conditions, further photoactivation resulted in the appearance of aggregates. Thus, when the closed‐ring fluorescent isomers were generated by photolysis and applied for staining, no aggregation and superior labeling were observed (Figures 3 and S1). These figures show that both probes undergo a very limited number of switching cycles. Thus, the switching kinetics in mitochondria are much slower than in solution (>2600 cycles in acetonitrile; Table 1), and switching was accompanied by bleaching. In general, the “reversibility” of all probes in mitochondria or attached to proteins is worse than that in solution, and this limits their use in superresolution microscopy.

### 2.4 Two‐Color Fluorescence Imaging

**FIGURE 4:**
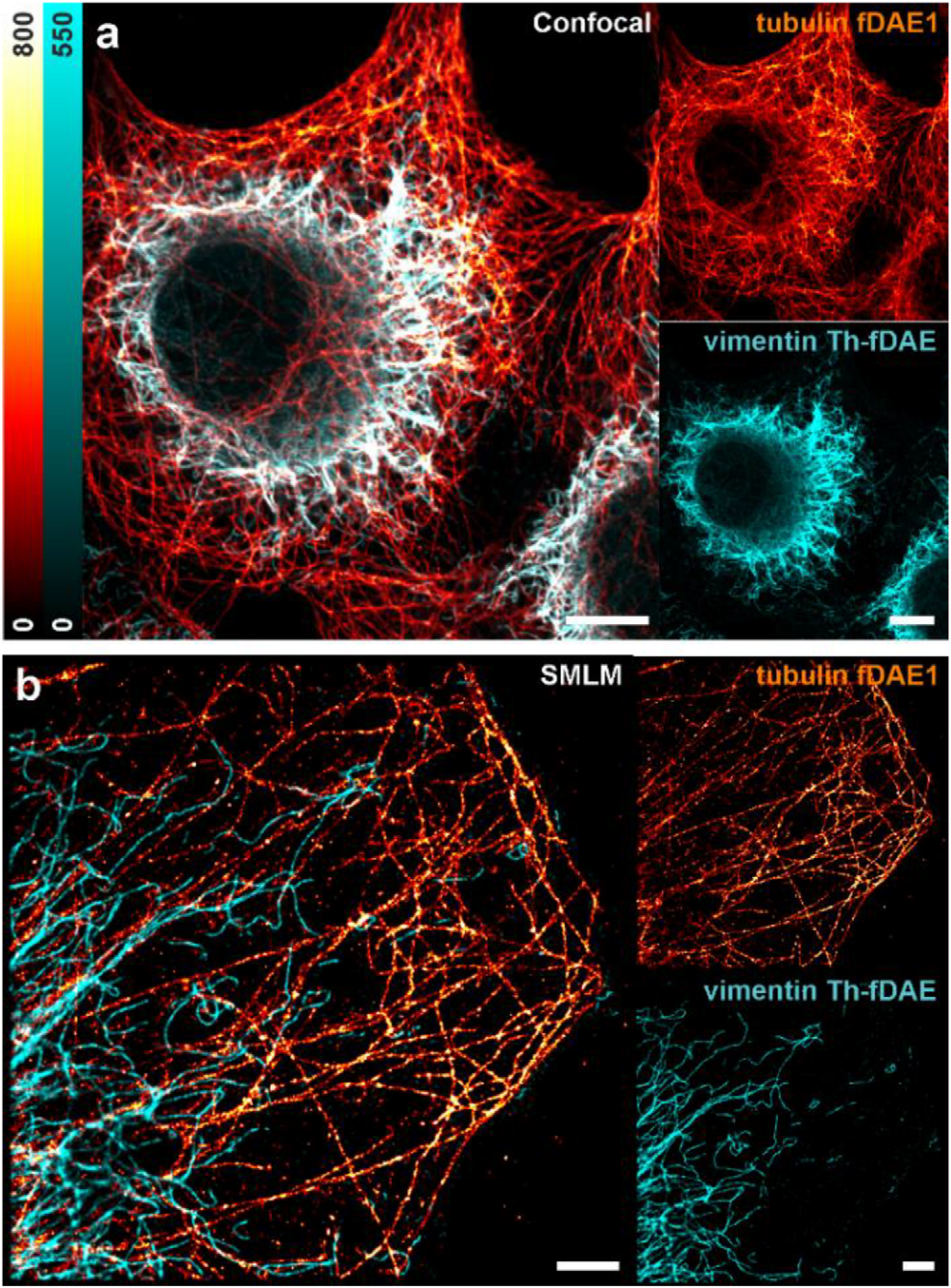
Indirect immunofluorescence images of COS7 cells. Tubulin filaments were labeled with **HO‐fDAE 1** attached to anti‐mouse (magenta), and the protein TOM20 in mitochondria was labeled with **Th‐fDAE** on anti‐rabbit secondary antibodies (cyan). Confocal (a) and single‐molecule localization microscopy (SMLM; b) images; scale bars: 10 µm.

The pair of **HO‐fDAE 1** and **Th‐fDAE** represents two photoactivatable dyes with strong emission and increased Stokes shifts (Table 1). This combination of properties is rare, and it enables multicolor and multimodal (with photoactivation option) imaging schemes. The spectral parameters (Table 1 and Figure 1) and different activation wavelengths (Figure 1) allowed us to obtain “true” separation of two colors (Figure 4) with low crosstalk, even when the detection windows were kept broad (Figure 1).

This and the relatively high “activation brightness” (product of the absorption coefficient at the activation wavelength and the ring‐closure quantum yield) led to the acquisition of strong signals in both channels. The absence of crosstalk is particularly important because it provides an additional “degree of freedom,” enables multicolor imaging (adding of the third color), and colocalization of biological objects without using any linear unmixing schemes, which require additional experiments and complicate the acquisition and data processing. We chose the pair **HO‐fDAE 1** and **Th‐fDAE** (Scheme 1, Figure 1, and Table 1) for labeling the cellular structures, followed by imaging by means of confocal and single molecule localization microscopies (SMLM) (Figures 3 and S2). Due to the lack of cell permeability of **Th‐fDAE**, two proteins were labelled by indirect immunofluorescence using secondary antibodies conjugated with compounds **HO‐fDAE 1** and **Th‐fDAE** (via *N*‐hydroxysuccinimidyl esters). Figures 4 and S2 show images of the tubulin fibers and the protein TOM20 in mitochondria of COS7 cells. As can be seen, the color separation is quite satisfactory, and the detection well beyond 515 nm minimizes the input of the background fluorescence of the cellular structures.

## 3 Conclusion

We synthesized “turn‐on” (green‐emitting) fDAEs with four hydroxyls and one carboxylic acid groups (HO‐fDAEs). The absence of the negative charges (in conjugates involving COOH group of the probe) facilitates cell permeability. The presence of polar hydroxyl groups makes the probes more soluble in aqueous buffers. HO‐fDAEs were used for the preparation of cell‐permeant mitochondrion‐selective probes and bioconjugation with proteins. We revealed the advantages of HO‐fDAEs based on high gain in the fluorescence signal, good photoactivation and fluorescence quantum yields, large Stokes shifts, and cell permeability. We combined **HO‐fDAE 1** in bio‐imaging with irreversibly photoactivatable thiophene‐substituted **Th‐fDAE** emitting red light. Apart from clean color separation, this pair provides an option to acquire images with optical superresolution based on single‐molecule switching algorithms. The limitations of the probes relate to the low number of switching cycles in aqueous buffers (unlike carboxylated fDAEs undergoing thousands of cycles [48]) and in conjugates with proteins. The low switching performance of fDAEs as bio‐conjugates with proteins remains the main obstacle for breakthroughs in using them as photoswitchable fluorescent markers in biology‐related superresolution microscopy [41, 43, 46]. However, the presence of charged groups in the structures of fDAEs, or combining fluorescent probes (not only fDAEs) as guests with macrocyclic hosts may, improve their properties; in particular, increase fluorescent quantum yields and brightness, enhance photostability, biocompatibility, switching performance, and photoresistance [52, 53]. Still, the usability of photoactivatable and photoswitchable fluorescent markers in advanced applications, like optical nanoscopy, is restricted, and this field requires further innovations. The results on photophysical properties and (two‐color) imaging presented here will contribute to a better understanding of the factors influencing the performance, applicability, and perspectives of photoswitchable fDAEs in bioconjugation techniques and fluorescence microscopy.

## Conflicts of Interest

The authors declare no conflict of interest

## Supporting information




**Supporting file 1**: chem70612‐sup‐0001‐SuppMat.docx
