# Supplementary material for: Fluorescent Diarylethenes With Polar Groups: Synthesis, Spectra, and Optical Microscopy Applications
Source: Chemistry. 2025 Dec 24;32(30):e03087. doi: 10.1002/chem.202503087 (PMC13472355; doi:10.1002/chem.202503087)
Supplement: Supplementary file 1 [file CHEM-32-e03087-s001.docx]

Supporting Information

**Fluorescent Diarylethenes with Polar Groups: Synthesis, Spectra, and Optical Microscopy Applications**

Kakishi Uno,^[a]^ Sven Nagorny,^[a]^ Ayse Aktalay,^[b]^ Dojin Kim,^[a]^ Mariano L. Bossi,*^[b]^

Vladimir N. Belov,*^[a]^ and Stefan W. Hell*^[a,b]^

[a]	Department of NanoBiophotonics, Max Planck Institute for Multidisciplinary Sciences (MPINAT), Am Fassberg 11, 37077 Göttingen, Germany

E-mail: [vladimir.belov@mpinat.mpg.de](mailto:vladimir.belov@mpinat.mpg.de), Stefan.Hell@mpinat.mpg.de

[b]	Department of Optical Nanoscopy, Max Planck Institute for Medical Research, Jahnstrasse 29, 69120 Heidelberg, Germany

E-mail: mariano.bossi@mr.mpg.de

**Table of contents**

Abbreviations chemistry	3

Photoswitching conditions	3

Cell culture	4

Antibodies and fluorescent probes	4

Staining of mitochondria	4

Two-color sample preparation	4

Conjugation with antibodies via amino groups	5

Confocal images and switching experiments on cells	5

Two-color SMLM Imaging	6

Synthesis description	8

Copies of NMR spectra	21

Methods and results of DFT calculations (structures and orbitals)	42

**Abbreviations chemistry**

Acetonitrile (MeCN), argon (Ar), bis(pinacolato)diboron (Bpin)_2_, bovine serum albumin (BSA), (diacetoxyiodo)benzene (DAIB), diarylethene (DAE), dichloromethane (DCM), dimethyl sulfoxide (DMSO), equivalent (equiv.), electrospray ionization (ESI), ethyl acetate (EA), fluorescent diarylethene (fDAE), *n*-hexane (n-Hex), high resolution mass-spectrometry (HRMS), methanol (MeOH), *N,N-*dimethylformamide (DMF), nuclear magnetic resonance (NMR), open form (OF), palladium (II) 1,1′-bis[(diphenylphosphino)ferrocen] dichloride (Pd(dppf)Cl_2_), bis(dibenzylideneacetone)palladium(0) (Pd(dba)_2_), phosphate buffer saline (PBS), photostationary state (PSS), potassium acetate (KOAc), reversed phase (RP), room temperature (r.t.), 2-dicyclohexylphosphino-2',6'-dimethoxybiphenyl (Sphos), thin layer chromatography (TLC), 2,2,6,6-tetramethylpiperidinyloxyl (TEMPO), trifluoroacetic acid (TFA), ultraviolet (UV), visible (Vis), volume ratio of two solvents (v/v).

**Photoswitching conditions**

Irradiation experiments were performed in a home-made setup described previously.^[1]^ It contained two high power light emitting diodes (LED; Thorlabs, M365L2 and M470L3) as irradiation sources, and a fiber-coupled diode array spectrometer (Ocean Optics, Flame-S UV-Vis configuration). For absorption measurements, a fiber-coupled halogen/deuterium light source (Ocean Optics, DH-2000-BAL) was used at 180 degrees. The sample was placed into a quartz cuvette for fluorescence (Hellma Analytics, 119F-10-40), in a peltier-based temperature-controlled cuvette holder, with four optical ports and magnetic stirring (Quantum Northwestern, Luma 40). A set of lenses and circular apertures was used to ensure the irradiation beams are collimated and have a diameter of around 6 mm at the sample. The collimation of the optical fibers at the sample holder was achieved by the use of dedicated achromatic lenses (Ocean Optics, 74-UV).

Absorption and emission of the sample were probed after each irradiation step, with the shutters of the irradiation sources closed. For fluorescence excitation, the irradiation source (470 nm) was switched on for ca. 10 microseconds. This pulse is much shorter than irradiation times (they are on the order of seconds). It was confirmed for all compounds that the excitation dose did not produce an observable change in the state of the photochromic system. Solutions in acetonitrile or aqueous buffer (concentrations 1-5 μM) were applied for switching experiments and calculations of the isomerization quantum yields. For irradiation experiments combined with HPLC measurements (i.e. distribution of photoproducts and calculation of the conversion degree in the PSS), higher concentrations of up to 15 μM were used in acetonitrile.

**Cell Culture**

COS7 cells (Cell Lines Service, 665470) were cultivated in an incubator (37°C, 5% CO_2_, 95% relative humidity). in DMEM:Ham's F12 medium (Gibco, 11320074) supplemented with fetal bovine serum (FBS) (ThermoFisher, 10500064) and 1% penicillin-streptomycin (Gibco, 15140122). For dissociation of cells, TrypLE Express (Gibco, 12604013) was used. Live-imaging experiments were performed in PBS as mounting media to reduce lipophilic interactions. Measurements times did not exceed 30 min for cell survival.

#### Antibodies and Other Fluorescent Conjugates

| **Product Name** | **Company** | **Catalog Number** |
| --- | --- | --- |
| anti-alpha tubulin clone 3A2 mouse | Synaptic Systems | 302211 |
| anti-vimentin EPR3776 rabbit | Abcam | ab92547 |
| anti-TOMM20 rabbit | Abcam | ab186735 |
| anti-clathrin rabbit | Abcam | ab21679 |
| unconjugated AffiniPure goat anti-rabbit | Jackson ImmunoResearch | 111-005-003 |
| unconjugated AffiniPure goat anti-mouse | Jackson ImmunoResearch | 115-005-003 |
| Hoechst 33342 | Biomol | CDX-B0030-M025 |
| MitoTracker DeepRed | ThermoFisher | M22426 |

**Staining of Mitochondria in Living Cells**

Live COS7 cells were stained with **HO-fDAE 3** (5 µM in DMEM (Gibco, 31966021) or PBS for experiments with the pre-irradiated compound) for 30 min in a cell culture incubator at 37°C. Cells were washed twice with PBS for 5 min. For colocalization experiments MitoTracker Deep Red (MT-DR) (100 nM) and Hoechst 33342 (5 µM) were added in the last washing step prior to imaging.

**Two-Color Sample Preparation**

COS7 cells were grown for 12–72 h on glass coverslips and then washed twice with PBS. Coverslips were placed on ice and treated with methanol (MeOH) previously cooled to -20 °C for 5 min, and finally washed twice with PBS. To reduce unspecific binding blocking buffer (2% bovine serum albumin (BSA) in PBS) was added and incubated for 15–30 min at room temperature. The coverslips were overlaid with the primary antibody solution prepared in a 1:1 dilution of blocking buffer in PBS and incubated in a humid chamber for 60–90 min at room temperature, or overnight at 4 °C. Next, the coverslips were washed with PBS (3×5 min). Coverslips were overlaid with the secondary antibody solution prepared in a 1:1 dilution of blocking buffer and PBS and incubated in a humid chamber for 60–90 min at room temperature. Finally, coverslips were washed with PBS (3×5 min) and mounted in PBS for imaging.

**Conjugation with antibodies via amino groups**

The pH of the unconjugated secondary antibody solution (2.4 mg/l) was adjusted to pH≈8 by the addition of aqueous NaHCO_3_ (1 M) to a final concentration of 100 mM. A concentrated solution (4–5 mM) of the dye in DMF was prepared. The NHS-ester derivative was added to the antibody solution at a dye to antibody ratio of 5:1 to 10:1 and stirred for 1 h at room temperature. The labelled antibody was purified by size exclusion chromatography with a pre-packed Sephadex G 25 column (PD-MiniTrap G25, GE Healthcare). The labelling was confirmed by UV Vis spectroscopy.

**Confocal Imaging and Switching Experiments on Cells**

Confocal images were acquired on an Abberior STED microscope (Expert Line, Abberior Instruments) at the Optical Microscopy Facility of the Max Planck Institute for Medical Research. The microscope was equipped with 355 nm, 405 nm, 485 nm, 561 nm and 640 nm excitation lines, and doughnut-shaped 488 nm, 595 nm and 775 nm depletion lines, a UPlanSApo 100x/1.40 oil 8/0.17/FN26.5 lens, an auto-focus system, and a spectral detection system. Images were acquired pixel by pixel with 80x80 nm pixel size, with excitation wavelengths and emission windows depending on the sample properties as indicated below.

Images were acquired in three channels: Hoechst channel via excitation with the355 nm laser (0.6 µW, 20 µs) and a detection window of 420–475 nm, which also functions as the activation channel, diarylethene channel with excitation by the 485 nm laser (30–40 µW, 20 µs) and a detection window of 515–620 nm and lastly the MT-DR channel with the 640 nm excitation laser (3 µW, 20 µs) and a detection window of 650–750 nm.

Photoswitching experiments on mitochondria were performed with the 485 nm excitation laser (30–40 µW, 20 µs) for probing the (initial) on-state, followed by irradiation with the 485 nm excitation laser (30–40 µW, 20 µs) until a constant level of low signal was reached (on the 6–10th frame) corresponding to the off-state. The probe was then activated with the 355 nm activation laser (20 mW, 100 µs), followed by imaging with the with 485 nm excitation laser (30–40 µW, 20 µs) for probing the on-state. The procedure was repeated until the fluorescence signal in mitochondria became dim.

Images of two-color samples were acquired sequentially, pixel by pixel, with the following sequence: first, an image in the channel of the phenyl-substituted diarylethene **HO-fDAE 1** was acquired by activation with the 355 nm laser (0.6 µW, 20–40 µs), excitation by the 485 nm (40–50 µW, 20–40 µs) laser and a detection window of 515–580 nm with three line accumulations, next, an image in the channel of the thienyl-substituted diarylethene **Th-fDAE** was acquired by activation with 405 nm laser (0.6 µW, 30 µs), excitation by the 561 nm (40–50 µW, 200–300 µs) laser and a detection window of 580–750 nm.

**SMLM Imaging of Two-Color Samples**

SMLM images were acquired on a custom-built SMLM setup equipped with a 405 nm (300 mW) activation line, 474 nm (500 mW) and 560 nm (1 W) excitation lines, a back illuminated EMCCD camera (Andor iXon 897 / 512×512 sensor), and a Leica HCX PL APO CS 100x/1.46 oil lens. Emission light was separated from the excitation and activation light with dichroic mirrors and filtered with emission filters: orange channel (580 nm (Semrock HC R561) / 589–739 nm (Semrock 665/150 BL HC)) and green channel (505 nm (Chroma T 505 lpxr) / 508–598 nm (Semrock 550/88 BL HC)). The measurements were set up and performed via a custom LabView (2019 32 bit) software, and the camera was controlled with software (Andor Solis), provided by the manufacturer.

Images of two channels were acquired sequentially with the orange and green filter cubes using 560 and 473 nm excitation lasers with powers of 160–180 and 20–25 mW (measured in the back focal plane of the objective lens), respectively. A 20 ms exposure time was used for all images. The 561 nm excitation laser power was selected sufficiently high to induce activation by Urbach-tail effect, hence, the imaging in the orange channel was performed without 405 nm activation laser. The 405 nm activation laser was switched on for imaging in the green channel between frames (with 500 μs pulses) with gradually increasing power until the acquisition was complete and the events became sparse.

SMLM images were processed using ImageJ (1.53t) with the ThunderSTORM plug-in. Images were filtered using a wavelet filter (B-spline) with an order of 3 and a scale of 2. The local maximum approach with 8-connected neighborhoods and an intensity threshold of 1.4–2.0 standard deviations of the 1st wavelet. An integrated Gaussian PSF model, the maximum likelihood fitting method with a fitting radius of 3 pixels, and an initial sigma value of 1.6 pixels were selected for sub-pixel localization of single molecules. For post-processing, a drift correction was applied based on cross correlation with a bin size according to the acquired number of frames. The trajectory smoothing factor was adjusted to minimize artificially harsh corrections. Sigma values were filtered to converge to a normal Gaussian distribution function separately for each channel. Photon numbers and uncertainty values were restricted (to 100 < intensity < 20000 and uncertainty < 45) to filter any outliers. Next, localizations were merged within the size of 0.5 pixel with 0 off-frames allowed. Lastly, a density filter was applied to remove noise caused by isolated localizations without at least 5 neighboring localizations in 100 nm proximity.

**Figure S1.** Mitochondria probe based on the fast-switching fDAEs with four hydroxyl groups and ethyl residue (**HO-fDAE 2**-TPP). Labelling was performed with the closed-ring fluorescent isomers. Note the limited number of switching cycles.

**Figure S2.** Two-color confocal and SMLM images of fixed COS7 cells. Tubulin filaments were stained with **HO-fDAE1** (COOH) - secondary antibody conjugates (red hot), the TOMM20 mitochondrial protein and clathrin pits were stained with **Th-fDAE**-secondary antibody conjugates (cyan). Scale bars: (a) 10 µm, (c) 5 µm, (b, d) 2.5 µM.

**Synthesis**

**High performance liquid chromatography (HPLC)**

Preparative HPLC was performed on puriFlash 4250 2X preparative HPLC/Flash hybrid system (Interchim) with a 2 mL injection loop, a 200-600 nm UV-Vis detector and an integrated ELSD detector. Preparative column: Interchim Uptisphere Strategy C18-HQ, 10 µm, 250×21.2 mm (US10C18HQ-250/212, Interchim), flow rate 20 mL/min, unless specified otherwise. Analytical TLC was performed on Merck Millipore ready-to-use plates with silica gel 60 and UV-indicator (F254). Flash chromatography was performed on Biotage Isolera 3.0 flash purification system using cartridges with regular SiO_2_ and solvent gradients indicated in the description of the synthesis. Analytical HPLC was performed on a KNAUER Azura system with a photodiode array detector, a 20 μL injection loop, and a 150 × 4 mm column (Knauer, Eurospher II 100-10 C18A with precolumn, Vertex Plus), at a flowrate of 1.2 mL/min with water/MeCN gradient; and both solvents containing 0.1% v/v of TFA.

**Nuclear magnetic resonance (NMR) spectroscopy**

NMR Spectra (^1^H, ^13^C and ^19^F) were recorded on an *Agilent 400MR DD2* spectrometer (400 MHz ^1^H, 101 MHz ^13^C) or Bruker Avance III HD 500 (500 MHz ^1^H, 125 MHz ^13^C). All ^1^H- and ^13^C-NMR spectra are referenced to the signals of the residual protons and ^13^C in CD_3_OD (^1^H: 3.35 ppm, ^13^C: 49.30 ppm), [d_7_]DMF (^1^H: 8.03 ppm, ^13^C: 163.2 ppm). Multiplicities of the signals are described as follows: s = singlet, d = doublet, t = triplet, q = quartet, quin = quintet, sext = sextet, sept = septet, m = multiplet (overlap), br = broad. Coupling constants (*J*) are given in Hz. The FIDs were processed with the software MestReNova.

**ESI and high-resolution mass-spectrometry (ESI-MS)**

ESI-MS were recorded on a Varian 500-MS spectrometer (Agilent). ESI-HRMS were recorded on a MICROTOF spectrometer (Bruker) equipped with an *Apollo* ion source and a direct injector as an LC-autosampler (Agilent RR 1200).

Compound **A΄:** To a solution of sodium hydride (NaH) (109 mg, 4.5 mmol, 1.2 equiv.) in DMF (5 mL) was slowly added 2,2-dimethyl-1,3-dioxolane-4-methanol (500 mg, 3.8 mmol, 1 equiv.), which was then stirred for 30 min at 40 ℃. A solution of 4-bromobenzyl bromide (946 mg, 3.8 mmol, 1 equiv.) in 1 mL DMF was slowly added to the solution, and the solution was stirred overnight at room temperature. After the reaction was complete, the saturated NH_4_Cl solution was added, and the product was extracted with diethyl ether. The organic layer was dried over anhydrous Na_2_SO_4_ and concentrated under reduced pressure. The crude material was subjected to chromatography on silica gel with gradient elution (EA/n-Hex: 10/90 → 50/50) to yield 741 mg (65%) of **A΄** as colorless oil.

^1^H-NMR (400 MHz, CD_3_OD): δ (ppm) = 7.45 (d, *J* = 8.3 Hz, 2H), 7.22 – 7.17 (m, 2H), 4.56 – 4.46 (m, 2H), 4.32 – 4.25 (m, 1H), 4.04 (dd, *J* = 8.3, 6.4 Hz, 1H), 3.72 (ddd, *J* = 8.3, 6.3, 0.6 Hz, 1H), 3.56 – 3.43 (m, 2H), 1.41 (d, *J* = 0.9 Hz, 3H), 1.35 (t, *J* = 0.7 Hz, 3H). ^13^C NMR (101 MHz, CD_3_OD): δ (ppm) = 137.0, 131.5, 129.3, 121.5, 109.4, 74.7, 72.7, 71.2, 66.7, 26.8, 25.4.

HRMS (ESI+, *m/z*): [M+Na]^+^ calcd for C_13_H_17_BrNaO_3_^+^, 323.0253; found, 323.0245.

Compound **A:** Compound **A΄** (500 mg, 1.7 mmol, 1 equiv.), (BPin)_2_ (675 mg, 2.7 mmol, 1.6 equiv.), Pd(dppf)Cl_2_ (243 mg, 0.3 mmol, 0.2 equiv.), KOAc (489 mg, 5.0 mmol, 3 equiv.) were dissolved in anhydrous dioxane (15 mL), and the reaction mixture was purged with Ar. The mixture was heated to 90 ℃ and stirred for 12 h. The reaction mixture was poured into brine, and extracted with DCM. The organic solution was dried over Na_2_SO_4_. After concentrating under reduced pressure, the crude material was subjected to chromatography on silica gel with gradient elution (EA/n-Hex: 0/100 → 20/80) to yield 530 mg (92%) of **A** as a white powder.

^1^H-NMR (400 MHz, CD_3_OD): δ (ppm) = 7.80 (d, *J* = 8.1 Hz, 2H), 7.34 (d, *J* = 8.1 Hz, 2H), 4.65 – 4.55 (m, 2H), 4.34 – 4.26 (m, 1H), 4.06 (dd, *J* = 8.3, 6.4 Hz, 1H), 3.76 – 3.70 (m, 1H), 3.55 (dd, *J* = 9.8, 5.7 Hz, 1H), 3.47 (dd, *J* = 9.8, 5.4 Hz, 1H), 1.44 – 1.41 (m, 3H), 1.38 – 1.36 (m, 3H), 1.35 (s, 12H). ^13^C NMR (101 MHz, CD_3_OD): δ (ppm) = 141.3, 135.0, 127.0, 109.6, 83.9, 74.9, 73.5, 67.0, 26.9, 25.6, 25.0.

HRMS (ESI+, *m/z*): [M+Na]^+^ calcd for C_19_H_29_BNaO_5_^+^, 370.2037; found, 370.2042.

Compound **B΄:** To a solution of 164 mg of 60% sodium hydride (NaH) (99 mg, 4.1 mmol, 2.4 equiv.) in anhydrous DMF (5 mL) was slowly added 2-(tetrahydro-2*H*-pyran-2-yloxy)ethanol (500 mg, 3.4 mmol, 2 equiv.), which was then stirred for 30 min at 40 ℃. A solution of 1-bromo-3,5-bis(bromomethyl)benzene (586 mg, 1.71 mmol, 1 equiv.) in 1 mL DMF was slowly added to the solution, and the solution was stirred overnight at room temperature. After the reaction was complete, the saturated NH_4_Cl solution was added, and the product was extracted with diethyl ether. The organic layer was dried over anhydrous Na_2_SO_4_ and concentrated under reduced pressure. The crude material was subjected to chromatography on silica gel with gradient elution (EA/n-Hex: 10/90 → 50/50) to yield 417 mg (52%) of **B΄** as colorless oil.

^1^H-NMR (400 MHz, CD_3_OD): δ (ppm) = 7.45 – 7.40 (m, 2H), 7.23 – 7.20 (m, 1H), 4.64 (t, *J* = 3.6 Hz, 2H), 4.56 – 4.52 (m, 4H), 3.91 – 3.84 (m, 4H), 3.67 – 3.60 (m, 6H), 3.53 – 3.47 (m, 2H), 1.90 – 1.80 (m, 2H), 1.78 – 1.69 (m, 2H), 1.67 – 1.49 (m, 8H). ^13^C NMR (101 MHz, CD_3_OD): δ (ppm) = 141.0, 129.6, 125.1, 122.7, 99.1, 72.3, 69.8, 66.8, 62.4, 30.7, 25.6.

HRMS (ESI+, *m/z*): [M+Na]^+^ calcd for C_22_H_33_BrNaO_6_^+^, 495.1353; found, 495.1345.

Compound **B:** Compound **B΄** (415 mg, 0.88 mmol, 1 equiv.), (BPin)_2_ (356 mg, 1.4 mmol, 1.6 equiv.), Pd(dppf)Cl_2_ (128 mg, 0.18 mmol, 0.2 equiv.), KOAc (258 mg, 2.6 mmol, 3 equiv.) were dissolved in anhydrous dioxane (10 mL), and the reaction mixture was purged with Ar. The mixture was heated to 90 ℃ and stirred for 12 h. The reaction mixture was poured into brine, and it was extracted with DCM. The organic solution was dried over Na_2_SO_4_. After concentrating under reduced pressure, the crude material was subjected to chromatography on silica gel with gradient elution (EA/n-Hex: 0/100 → 30/70) to yield 405 mg (90%) of **B** as colorless oil.

^1^H-NMR (400 MHz, CD_3_OD): δ (ppm) = 7.69 (d, *J* = 1.3 Hz, 2H), 7.48 (s, 1H), 4.66 – 4.62 (m, 2H), 4.62 – 4.53 (m, 4H), 3.91 – 3.83 (m, 4H), 3.67 – 3.59 (m, 6H), 3.52 – 3.46 (m, 2H), 1.85 (qt, *J* = 9.2, 4.2 Hz, 2H), 1.77 – 1.68 (m, 2H), 1.67 – 1.48 (m, 8H), 1.33 (s, 12H). ^13^C NMR (101 MHz, CD_3_OD): δ (ppm) = 138.0, 133.6, 130.2, 99.0, 83.9, 73.2, 69.6, 66.8, 62.3, 25.6, 25.0, 19.6.

HRMS (ESI+, *m/z*): [M+Na]^+^ calcd for C_28_H_45_BNaO_8_^+^, 543.3105; found, 543.3109.

Compound **C(Et):** Compound **E** was prepared according to the literature procedure (see Scheme above). A solution of triphenylphosphine (26 mg, 99 µmol, 1.7 equiv.) and I_2_ (25 mg, 99 µmol, 1.7 equiv.) in DCM (1 mL) was stirred at 35 ℃ for 10 min. Imidazole (11 mg, 163 µmol, 2.8 equiv.) was added to the mixture, and the mixture was stirred for 10 min. Compound **E** (50 mg, 58 µmol, 1 equiv.) was added to the mixture and the resulting mixture was stirred at 35 ℃ for 2h. The mixture was poured into saturated Na_2_S_2_O_5_ aqueous solution, and extracted with DCM. The organic solution was dried over Na_2_SO_4_. After concentrating under reduced pressure, the crude material was subjected to chromatography on silica gel with gradient elution (EA/n-Hex: 0/100 → 15/85) to yield 44 mg (78%) of **C(Et)** as a yellow green powder.

^1^H-NMR (400 MHz, CD_3_OD, mixture of *syn*- and *anti*-isomers): δ (ppm) = 8.08 (dd, *J* = 4.3, 1.5 Hz, 1.3H), 8.02 – 7.94 (m, 2H), 7.81 – 7.74 (m, 0.7H), 6.99 – 6.91 (m, 1.3H), 6.83 – 6.76 (m, 0.7H), 3.19 (t, *J* = 6.5 Hz, 0.7H), 3.09 – 2.91 (m, 1.3H), 2.66 – 2.21 (m, 4H), 1.96 – 1.43 (m, 4H), 1.39 (t, *J* = 7.6 Hz, 1H), 1.02 (t, *J* = 7.6 Hz, 2H). ^13^C NMR (101 MHz, CD_3_OD): δ (ppm) = 148.3, 146.4, 143.0, 142.9, 142.6, 142.5, 137.1, 137.0, 136.9, 136.8, 131.6, 131.5, 128.8, 128.7, 124.2, 124.2, 124.13, 124.10, 123.8, 123.70, 123.66, 123.60, 122.9, 96.9, 96.8, 96.7, 96.1, 34.8, 33.5, 33.4, 27.8, 27.1, 25.4, 24.9, 24.8, 19.5, 19.2, 12.0, 11.6, 4.4, 4.3. ^19^F NMR (376 MHz, CD_3_OD): δ (ppm) = –109.4 (m, 4F), –132.2 (m, 2F).

HRMS (ESI+, *m/z*): [M+Na]^+^ calcd for C_27_H_19_F_6_I_3_NaO_4_S_2_^+^, 988.7655; found, 988.7649.

Compound **D(Et):** A flask was charged with compound **C(Et)** (200 mg, 0.21 mmol, 1 equiv.), compound **A** (180 mg, 0.52 mmol, 2.5 equiv.), Pd(dba)_2_ (30 mg, 52 µmol, 0.25 equiv.), and SPhos (21 mg, 52 µmol, 0.25 equiv.). THF (15 mL) and saturated aqueous K_2_CO_3_ (15 mL) were added, and the mixture was degassed by Ar bubbling for 10 min. The solution was stirred at 80 ℃ for 30 min, while monitoring the progress of the reaction by TLC. After the reaction was complete, the mixture was poured into brine and extracted with DCM. The organic solution was dried over Na_2_SO_4_ and concentrated under reduced pressure. The crude material was subjected to chromatography on silica gel with gradient elution (EA/n-Hex: 5/95 → 30/70) to yield 181 mg (76%) of **D(Et)** as pale yellow powder.

^1^H-NMR (400 MHz, CD_3_OD, mixture of *syn*- and *anti*-isomers): δ (ppm) = 7.98 (dd, *J* = 4.4, 1.5 Hz, 1.3H), 7.88 (s, 0.7H), 7.87 – 7.80 (m, 1.3H), 7.63 – 7.56 (m, 3.3H), 7.52 – 7.44 (m, 3.9H), 7.44 – 7.38 (m, 1.3H), 7.33 – 7.27 (m, 1.3H), 7.21 (dd, *J* = 12.6, 7.8 Hz, 0.7H), 4.69 – 4.56 (m, 4H), 4.38 – 4.28 (m, 2H), 4.11 – 4.03 (m, 2H), 3.80 – 3.72 (m, 2H), 3.63 – 3.48 (m, 4H), 3.22 (t, *J* = 6.2 Hz, 0.7H), 3.07 – 2.87 (m, 1.3H), 2.75 – 2.33 (m, 4H), 2.05 – 1.78 (m, 2H), 1.74 – 1.49 (m, 2H), 1.46 – 1.34 (m, 13H), 1.05 (t, *J* = 7.6 Hz, 2H). ^13^C NMR (101 MHz, CD_3_OD): δ (ppm) = 148.5, 146.5, 144.5, 144.4, 144.3, 144.2, 139.4, 139.3, 139.2, 137.5, 137.4, 136.7, 136.5, 136.4, 132.3, 132.2, 131.9, 131.8, 128.6, 128.5, 128.1, 128.0, 127.4, 127.3, 124.4, 123.5, 123.4, 123.3, 123.1, 123.00, 121.2, 121.1, 109.6, 74.9, 73.1, 71.5, 66.9, 33.6, 33.5, 28.2, 28.0, 26.9, 25.5, 25.0, 24.9, 19.6, 19.2, 12.2, 11.7, 4.6, 4.4. ^19^F NMR (376 MHz, CD_3_OD): δ (ppm) = –109.6 (m, 4F), –132.2 (m, 2F).

HRMS (ESI+, *m/z*): [M+Na]^+^ calcd for C_53_H_53_F_6_INaO_10_S_2_^+^, 1177.1921; found, 1177.1922.

Compound **D(Et)΄:** To a acetonitrile solution (5 mL) containing compound **D(Et)** (180 mg, 0.16 mmol, 1 equiv.), was added triphenyl phosphine (245 mg, 0.94 mmol, 6 equiv.). The solution was heated to 80 ℃ and stirred for 6 h. After the reaction was complete, the solution was concentrated under reduced pressure. The crude material was subjected to chromatography on silica gel with gradient elution (MeOH/DCM: 1/99 → 5/95) to yield 181 mg (82%) of **D(Et)΄** as yellow powder.

^1^H-NMR (400 MHz, DMF-d_7_, mixture of *syn*- and *anti*-isomers): δ (ppm) = 8.4 (dd, *J* = 12.1, 1.6 Hz, 1.2H), 8.3 (dd, *J* = 4.2, 1.7 Hz, 1.2H), 8.2 (dd, *J* = 8.1, 1.8 Hz, 0.8H), 8.1 (ddd, *J* = 8.1, 3.2, 1.7 Hz, 0.8H), 8.0 – 7.8 (m, 21H), 7.6 – 7.4 (m, 4H), 4.7 (s, 2.4H), 4.6 (s, 1.6H), 4.3 (dq, *J* = 17.7, 6.1 Hz, 2H), 4.1 – 4.0 (m, 2H), 3.8 – 3.7 (m, 2H), 3.6 – 3.5 (m, 4H), 2.9 – 2.5 (m, 2H), 2.3 – 1.7 (m, 4H), 1.7 – 1.4 (m, 2H), 1.4 – 1.3 (m, 15H), 1.0 (t, *J* = 7.6 Hz, 2H). ^13^C NMR (101 MHz, DMF-d_7_): δ (ppm) = 144.9, 144.8, 144.7, 144.6, 141.0, 140.9,137.9, 137.8, 137.7, 137.5, 137.4, 137.3, 137.2, 136.1, 135.1, 135.0, 134.9, 134.0, 133.6, 131.5, 131.4, 129.4, 129.3, 128.7, 128.6, 128.34, 128.27, 128.2, 126.0, 125.7, 124.9 124.3 124.2, 124.1, 124.0, 121.7, 121.6, 121.4, 120.4, 120.3, 119.5, 119.40, 109.9, 75.9, 73.4, 73.3, 72.6, 72.5, 67.5, 29.3, 29.1, 29.0, 28.8, 27.5, 27.4, 26.1, 26.0, 25.9, 23.5, 23.1, 22.2, 22.1, 20.0, 12.9, 12.4. ^19^F NMR (376 MHz, CD_3_OD): δ (ppm) = –109.2 (m, 4F), –130.6 (m, 2F).

HRMS (ESI+, *m/z*): M^+^ calcd for C_71_H_68_F_6_O_10_PS_2_^+^, 1289.3890; found, 1289.3899.

Compound **HO-fDAE 2 (TPP):** To a methanol solution (8 mL) containing compound **D(Et)΄** (135 mg, 0.10 mmol, 1 equiv.), was added pyridinium p-toluene sulfonate (105 mg, 0.42 mmol, 4 equiv.). The solution was heated to 65 ℃ and stirred for 16 h. After the reaction was complete, the mixture was poured into brine and extracted with EA. The organic layer was washed with brine and water. The organic solution was dried over Na_2_SO_4_ and concentrated under reduced pressure. The crude material was subjected to chromatography on silica gel with gradient elution (MeOH/DCM: 1/99 → 10/90) to yield 102 mg (73%) of **HO-fDAE 2 (TPP)** as yellow powder.

^1^H-NMR (500 MHz, DMF-d_7_, mixture of *syn*- and *anti*-isomers): δ (ppm) = 8.41 (dd, *J* = 14.1, 1.6 Hz, 1.2H), 8.31 (dd, *J* = 3.9, 1.6 Hz, 1.2H), 8.23 (dd, *J* = 8.1, 1.7 Hz, 0.8H), 8.05 (dd, *J* = 5.3, 1.8 Hz, 0.8H), 8.01 – 7.75 (m, 21H), 7.58 – 7.45 (m, 4H), 5.05 – 4.91 (m, 2H), 4.85 – 4.71 (m, 2H), 4.66 – 4.58 (m, 4H), 3.85 – 3.76 (m, 2H), 3.65 – 3.47 (m, 4H), 2.90 – 2.57 (m, 2H), 2.21 – 1.76 (m, 4H), 1.64 – 1.41 (m, 2H), 1.34 – 1.30 (m, 3.2H), 1.02 (t, *J* = 7.6 Hz, 1.8H). ^13^C NMR (126 MHz, DMF-d_7_): δ (ppm) = 174.3, 173.8, 173.6, 149.7, 148.1, 147.9, 144.9, 144.9, 144.8, 144.7, 141.5, 141.44, 141.40, 141.35, 137.7, 137.6, 137.5, 137.4, 137.3, 137.2, 136.2, 136.1, 135.1, 136.0, 135.0, 134.9, 134.8, 134.0, 133.6, 133.5, 131.5, 131.37, 131.3, 131.2, 129.3, 129.2, 129.0, 128.7, 128.5, 128.26, 128.3, 128.1, 127.0, 126.0, 125.7, 124.9, 124.1, 124.0, 121.7, 121.5, 121.4, 120.3, 120.2, 119.6, 119.5, 73.7, 73.6, 73.3, 73.2, 72.6, 72.5, 70.3, 65.4, 65.1, 65.0, 64.8, 63.1, 57.4, 57.1, 57.0, 54.9, 43.3, 43.2, 37.54, 37.45, 34.0, 33.6, 33.4, 33.0, 32.8, 32.6, 32.5, 29.3, 29.1, 29.0, 28.9, 28.8, 28.7, 28.0, 27.9, 27.7, 27.6, 27.4, 27.3, 27.2, 27.1, 26.9, 26.0, 25.9, 25.8, 24.6, 23.9, 23.5, 23.4, 23.4, 23.1, 22.1, 22.0, 21.7, 21.5, 20.2, 19.95, 19.9, 19.8, 14.8 14.7, 14.6, 12.9, 12.4, 12.0. ^19^F NMR (471 MHz, CD_3_OD): δ (ppm) = –109.7 (m, 4F), –130.6 (m, 2F).

HRMS (ESI+, *m/z*): M^+^ calcd for C_65_H_60_F_6_O_10_PS_2_^+^, 1209.3264; found, 1209.3262.

Compound **C(iBu):** Compound **E΄** was prepared according to the literature procedure (see Scheme above). A solution of triphenylphosphine (26 mg, 100 µmol, 1.7 equiv.) and I_2_ (25 mg, 100 µmol, 1.7 equiv.) in DCM (1 mL) was stirred for 10 min at room temperature. Imidazole (11 mg, 165 µmol, 2.8 equiv.) was added to the mixture, and the mixture was stirred for 10 min. Compound **E΄** (52 mg, 59 µmol, 1 equiv.) was added to the mixture and the resulting mixture was stirred for 2h. The mixture was poured into saturated Na_2_S_2_O_5_ aqueous solution, and extracted with DCM. The organic solution was dried over Na_2_SO_4_. After concentrating under reduced pressure, the crude material was subjected to chromatography on silica gel with gradient elution (EA/n-Hex: 0/100 → 10/90) to yield 53 mg (91%) of **C(iBu)** as a yellow green powder.

^1^H-NMR (400 MHz, CD_3_OD, mixture of *syn*- and *anti*-isomers): δ (ppm) = 8.09 (dd, *J* = 7.1, 1.4 Hz, 1.4H), 8.02 – 7.94 (m, 2H), 7.85 – 7.64 (m, 0.6H), 6.99 (ddd, *J* = 22.1, 8.1, 2.2 Hz, 1.4H), 6.92 – 6.63 (m, 0.6H), 3.19 (t, *J* = 6.7 Hz, 0.6H), 3.06 – 2.87 (m, 1.4H), 2.71 – 2.55 (m, 0.6H), 2.49 – 2.39 (m, 0.6H), 2.38 – 2.23 (m, 1.4H), 2.23 – 2.10 (m, 1.4H), 2.07 – 1.30 (m, 5H), 1.05 (d, *J* = 6.6 Hz, 0.9H), 0.95 (d, *J* = 7.4 Hz, 0.9H), 0.87 (d, *J* = 6.4 Hz, 2.1H), 0.76 (d, *J* = 6.5 Hz, 2.1H). ^13^C NMR (101 MHz, CD_3_OD): δ (ppm) = 147.0, 146.5, 142.9, 142.7, 142.6, 142.4, 137.0, 136.8, 131.6, 131.5, 131.5, 131.4, 128.9, 128.8, 128.6, 128.5, 124.81, 124.75, 124.3, 124.2, 124.0, 123.9, 123.7, 123.4, 123.3, 96.9, 96.7, 96.6, 35.2, 35.1, 34.8, 33.6, 33.4, 31.7, 29.8, 27.7, 27.1, 26.2, 26.1, 25.5 24.7, 23.8, 22.8, 21.9, 21.4, 14.3, 4.24, 4.21. ^19^F NMR (376 MHz, CD_3_OD): δ (ppm) = –109.2 (m, 4F), –132.6 (m, 2F).

HRMS (ESI+, *m/z*): [M+Na]^+^ calcd for C_29_H_23_F_6_I_3_NaO_4_S_2_^+^, 1016.7968; found, 1016.7965.

Compound **D(iBu):** A flask was charged with compound **C(iBu)** (80 mg, 0.80 µmol, 1 equiv.), compound **2** (70 mg, 201 µmol, 2.5 equiv.), Pd(dba)_2_ (12 mg, 20 µmol, 0.25 equiv.), and SPhos (8 mg, 20 µmol, 0.25 equiv.). THF (5 mL) and saturated aqueous K_2_CO_3_ (5 mL) were added, and the mixture was degassed by Ar bubbling for 10 min. The solution was stirred at 80 ℃ for 30 min, while monitoring the progress of the reaction by TLC. After the reaction was complete, the mixture was poured into brine and extracted with DCM. The organic solution was dried over Na_2_SO_4_ and concentrated under reduced pressure. The crude material was subjected to chromatography on silica gel with gradient elution (EA/n-Hex: 5/95 → 30/70) to yield 71 mg (75%) of **D(iBu)** as pale yellow powder.

^1^H-NMR (500 MHz, CD_3_OD, mixture of *syn*- and *anti*-isomers): δ (ppm) = 7.99 (dd, *J* = 12.3, 1.6 Hz, 1.3H), 7.87 (s, 0.7H), 7.86 – 7.80 (m, 1.3H), 7.69 – 7.55 (m, 3.3H), 7.53 – 7.29 (m, 7.3H), 4.69 – 4.55 (m, 4H), 4.37 – 4.27 (m, 2H), 4.11 – 4.04 (m, 2H), 3.80 – 3.72 (m, 2H), 3.63 – 3.48 (m, 4H), 3.22 (t, *J* = 6.9 Hz, 0.7H), 3.02 – 2.83 (m, 1.3H), 2.78 – 1.95 (m, 7H), 1.84 – 1.62 (m, 2H), 1.45 – 1.41 (m, 6H), 1.39 – 1.35 (m, 6H), 1.08 (d, *J* = 6.6 Hz, 1H), 0.99 (d, *J* = 6.3 Hz, 1H), 0.90 (d, *J* = 6.4 Hz, 2H), 0.76 (d, *J* = 6.5 Hz, 2H). ^13^C NMR (126 MHz, CD_3_OD): δ (ppm) = 147.5, 147.2, 146.7, 146.6, 144.5, 144.3, 144.1, 139.4, 139.3, 139.2, 139.0, 137.5, 137.4, 136.6, 136.4, 132.2, 132.0, 131.8, 131.7, 128.6, 128.5, 128.2, 127.9, 127.8, 127.5, 127.4, 127.3, 127.2, 124.9, 124.8, 124.4, 124.2, 124.1, 123.5, 123.4, 122.8, 121.2, 121.1, 121.0, 109.7, 109.6, 74.9, 73.2, 73.1, 73.0, 72.2, 71.5, 70.7, 66.9, 64.19, 64.15, 35.3, 35.1, 33.5, 31.1, 28.4, 27.9, 26.9, 26.23, 26.2, 25.5, 24.7, 23.8, 22.7, 22.0, 21.5, 21.4, 4.4, 4.3. ^19^F NMR (471 MHz, CD_3_OD): δ (ppm) = –109.0 (m, 4F), –132.5 (m, 2F).

HRMS (ESI+, *m/z*): [M+Na]^+^ calcd for C_55_H_57_F_6_INaO_10_S_2_^+^, 1205.2234; found, 1205.2210.

Compound **D(iBu)΄:** To an acetonitrile solution (2 mL) containing compound **D(iBu)** (50 mg, 42 µmol, 1 equiv.), was added triphenyl phosphine (78 mg, 296 µmol, 7 equiv.). The solution was heated to 80 ℃ and stirred for 6 h. After the reaction was complete, the solution was concentrated under reduced pressure. The crude material was subjected to chromatography on silica gel with gradient elution (MeOH/DCM: 1/99 → 5/95) to yield 55 mg (90%) of **D(iBu)΄** as yellow powder.

^1^H-NMR (500 MHz, DMF-d_7_, mixture of *syn*- and *anti*-isomers): δ (ppm) = 8.43 (dd, *J* = 14.2, 1.6 Hz, 1.2H), 8.31 – 8.26 (m, 2H), 8.11 – 8.07 (m, 0.8H), 8.00 – 7.73 (m, 21H), 7.58 – 7.44 (m, 4H), 4.68 (d, *J* = 3.6 Hz, 2.4H), 4.63 (d, *J* = 7.3 Hz, 1.6H), 4.37 – 4.27 (m, 2H), 4.13 – 4.05 (m, 2H), 3.77 – 3.71 (m, 2H), 3.64 – 3.54 (m, 4H), 2.90 – 2.45 (m, 2H), 2.43 – 1.44 (m, 7H), 1.37 – 1.22 (m, 14H), 1.04 – 0.79 (m, 6H). ^13^C NMR (126 MHz, DMF-d_7_): δ (ppm) = 148.2, 148.0, 147.9, 145.0, 144.9, 144.70, 144.66, 141.1, 141.0, 140.9, 137.9, 137.8, 137.7, 137.5, 137.3, 137.2, 136.2, 135.0, 134.9, 133.8, 133.6, 133.4, 131.5, 131.4, 129.4, 129.3, 128.8, 128.7, 128.5, 128.4, 128.3, 128.2, 126.4, 126.3, 126.2, 125.8, 125.70, 124.9, 121.8, 121.6, 121.4, 121.3, 120.1, 119.6, 119.4, 109.9, 75.9, 73.4, 73.3, 72.6, 72.5, 67.5, 27.7, 27.4, 27.1, 27.0, 26.5, 26.0, 25.8, 23.8, 23.5, 23.4, 23.1, 22.8, 22.2, 22.1, 21.7, 21.6, 19.4, 14.7. ^19^F NMR (376 MHz, CD_3_OD): δ (ppm) = –109.1 (m, 4F), –130.9 (m, 2F).

HRMS (ESI+, *m/z*): M^+^ calcd for C_73_H_72_F_6_O_10_PS_2_^+^, 1317.4203; found, 1317.4209.

Compound **HO-fDAE 3 (TPP):** To a methanol solution (1 mL) containing compound **D(iBu)΄** (45 mg, 31 µmol, 1 equiv.), was added pyridinium p-toluene sulfonate (39 mg, 156 µmol, 5 equiv.). The solution was heated to 70 ℃ and stirred for 6 h. After the reaction was complete, the mixture was poured into brine and extracted with EA. The organic layer was washed with brine and water. The organic solution was dried over Na_2_SO_4_ and concentrated under reduced pressure. The crude material was subjected to chromatography on silica gel with gradient elution (MeOH/DCM: 1/99 → 10/90) to yield 15 mg (35%) of **HO-fDAE 3 (TPP)** as yellow powder.

^1^H-NMR (500 MHz, DMF-d_7_, mixture of *syn*- and *anti*-isomers): δ (ppm) = 8.42 (dd, *J* = 13.7, 1.5 Hz, 1.2H), 8.31 – 8.26 (m, 2H), 8.08 (dd, *J* = 8.1, 1.7 Hz, 0.8H), 8.00 – 7.72 (m, 21H), 7.58 – 7.44 (m, 4H), 5.00 – 4.88 (m, 2H), 4.82 – 4.67 (m, 2H), 4.66 – 4.58 (m, 4H), 3.86 – 3.76 (m, 2H), 3.67 – 3.55 (m, 4H), 2.89 – 2.44 (m, 2H), 2.38 – 1.42 (m, 7H), 1.37 – 1.32 (m, 2H), 1.03 – 0.78 (m, 6H). ^13^C NMR (126 MHz, DMF-d_7_): δ (ppm) = 148.2, 148.0, 147.9, 145.1, 144.9, 144.8, 144.7, 141.5, 141.4, 141.3, 137.7, 137.6, 137.5, 137.4, 137.2, 136.1, 136.0, 135.1, 135.0, 134.9, 134.8, 133.60, 133.3, 131.5, 131.4, 131.3, 131.2, 129.3, 129.2, 129.1, 128.8, 128.4, 128.3, 128.2, 128.1, 126.4, 126.3, 126.2, 125.8, 125.7, 124.9, 121.7, 121.5, 121.3, 120.3, 120.1, 119.6, 119.4, 73.7, 73.6, 73.3, 73.2, 72.5, 72.4, 70.3, 65.4, 65.3, 65.0, 64.9, 63.1, 57.4, 57.0, 54.9, 43.2, 37.5, 37.4, 34.0, 33.6, 33.4, 33.3, 32.8, 32.6, 29.3, 29.1, 29.0, 28.7, 28.0, 27.6, 27.2, 27.1, 27.0, 26.9, 26.5, 25.8, 25.7, 24.6, 23.8, 23.5, 23.4, 23.1, 23.0, 22.7, 22.1, 21.9, 21.7, 21.5, 20.2, 19.8, 14.7, 14.6, 12.0. ^19^F NMR (471 MHz, CD_3_OD): δ (ppm) = –109.1 (m, 4F), –130.9 (m, 2F).

HRMS (ESI+, *m/z*): M^+^ calcd for C_67_H_64_F_6_O_10_PS_2_^+^, 1237.3577; found, 1237.3571.

Compound **F:** Compound **E** was prepared according to the literature procedure.^[S1]^ A flask was charged with compound **E** (200 mg, 0.23 mmol, 1 equiv.), compound **B** (303 mg, 0.58 mmol, 2.5 equiv.), Pd(dba)_2_ (34 mg, 58 µmol, 0.25 equiv.), and SPhos (24 mg, 58 µmol, 0.25 equiv.). THF (15 mL) and saturated aqueous K_2_CO_3_ (15 mL) were added, and the mixture was degassed by Ar bubbling for 10 min. The solution was stirred at 80 ℃ for 30 min, while monitoring the progress of the reaction by TLC. After the reaction was complete, the mixture was poured into brine and extracted with DCM. The organic solution was dried over Na_2_SO_4_ and concentrated under reduced pressure. The crude material was subjected to chromatography on silica gel with gradient elution (EA/n-Hex: 10/90 → 70/30) to yield 167 mg (51%) of **F** as yellow powder.

^1^H-NMR (400 MHz, CD_3_OD, mixture of *syn*- and *anti*-isomers): δ (ppm) = 7.98 (s, 1.4H), 7.91 (s, 0.6H), 7.89 – 7.80 (m, 1.4H), 7.71 – 7.60 (m, 0.6H), 7.56 – 7.48 (m, 2.8H), 7.45 – 7.26 (m, 4H), 7.26 – 7.14 (m, 1.2H), 4.71 – 4.54 (m, 12H), 3.95 – 3.78 (m, 8H), 3.75 – 3.37 (m, 18H), 2.74 – 2.28 (m, 4H), 1.89 – 1.39 (m, 28.9H), 1.05 (t, *J* = 7.6 Hz, 2.1H). ^13^C NMR (101 MHz, CD_3_OD): δ (ppm) = 148.3, 147.1, 144.5, 144.4, 144.3, 140.0, 139.9, 138.5, 138.4, 136.5, 132.6, 132.4, 132.1, 128.2, 127.3, 127.2, 125.6, 125.5, 125.4, 123.8, 123.4, 121.1, 99.2, 99.1, 72.9, 72.8, 69.8, 66.8, 62.5, 62.4, 62.3, 62.2, 62.0, 61.9, 32.7, 32.5, 30.7, 29.8, 25.9, 25.5, 23.8, 23.5, 19.8, 19.6, 19.2, 12.1, 11.7. ^19^F NMR (376 MHz, CD_3_OD): δ (ppm) = –109.7 (m, 4F), –132.2 (m, 2F).

HRMS (ESI+, *m/z*): [M+Na]^+^ calcd for C_71_H_86_F_6_NaO_17_S_2_^+^, 1411.5103; found, 1411.5075.

Compound **F΄:** Compound **F** (50 mg, 36 μmol, 1 equiv.) was dissolved in a mixture of ACN (700 μL) and water (300 μL). To the solution were added TEMPO (28 mg, 180 μmol, 5 equiv.) and DAIB (58 mg, 180 μmol, 5 equiv.), and the reaction mixture was stirred for 30 min, while monitoring the progress of the reaction by TLC. After the reaction was complete, the mixture was poured into water and extracted with EA. The organic solution was dried over Na_2_SO_4_ and concentrated under reduced pressure. The crude material was subjected to chromatography on silica gel with gradient elution (EA/n-Hex: 30/70 → 100/0) to yield 37 mg (73%) of **F΄** as yellow powder.

^1^H-NMR (400 MHz, CD_3_OD, mixture of *syn*- and *anti*-isomers): δ (ppm) = 8.00 – 7.96 (m, 1.4H), 7.93 – 7.81 (m, 2H), 7.69 – 7.62 (m, 0.6H), 7.61 – 7.49 (m, 2.8H), 7.47 – 7.27 (m, 4H), 7.25 – 7.14 (m, 1.2H), 4.72 – 4.57 (m, 12H), 3.99 – 3.81 (m, 8H), 3.77 – 3.44 (m, 16H), 2.76 – 1.96 (m, 6H), 1.90 – 1.33 (m, 26.9H), 1.11 – 1.04 (m, 2.1H). ^13^C NMR (101 MHz, CD_3_OD): δ (ppm) = 147.9, 144.3, 144.2, 140.1, 140.0, 139.9, 139.8, 138.5, 138.3, 136.4, 136.3, 132.8, 132.3, 128.0, 127.3, 125.4, 125.3, 125.2, 125.1, 124.0, 123.4, 123.1,121.0, 120.7, 99.6, 99.1, 99.0, 72.7, 72.5, 72.4, 69.8, 69.5, 69.4, 67.1, 66.7, 66.6, 62.8, 62.3, 37.0, 30.6, 30.5, 29.7, 25.4, 25.4, 25.2, 20.2, 19.7, 19.5, 19.4, 19.1, 15.9, 11.7. ^19^F NMR (376 MHz, CD_3_OD): δ (ppm) = –109.5 (m, 4F), –132.0 (m, 2F).

HRMS (ESI+, *m/z*): M^+^ calcd for C_71_H_84_F_6_NaO_18_S_2_^+^, 1425.4895; found, 1425.4958.

Compound **HO-fDAE 1 (COOH):** To a methanol solution (1 mL) containing compound **F΄** (35 mg, 25 µmol, 1 equiv.), was added pyridinium p-toluene sulfonate (50 mg, 251 µmol, 8 equiv.). The solution was heated to 70 °C and stirred for 3 h. Purification was carried out by RP column chromatography with gradient elution (0.1% aq. TFA / ACN, 75/25 → 0/100). Lyophilization yielded 13 mg (49%) of compound **HO-fDAE 1 (COOH)** as a yellow powder.

^1^H-NMR (500 MHz, DMF-d_7_, mixture of *syn*- and *anti*-isomers): δ (ppm) = 8.44 (dd, *J* = 23.1, 1.6 Hz, 1.3H), 8.33 (dd, *J* = 4.6, 1.6 Hz, 0.7H), 8.23 (ddd, *J* = 20.1, 8.1, 1.7 Hz, 1.3H), 8.10 – 8.06 (m, 0.7H), 7.98 (t, *J* = 6.5 Hz, 0.7H), 7.92 – 7.77 (m, 3.9H), 7.70 (d, *J* = 12.3 Hz, 1.4H), 7.51 (s, 1.3H), 7.45 (d, *J* = 4.3 Hz, 0.7H), 4.67 (d, *J* = 3.0 Hz, 5.2H), 4.60 (d, *J* = 5.4 Hz, 2.8H), 3.71 (t, *J* = 5.1 Hz, 5.2H), 3.68 – 3.65 (m, 2.8H), 3.63 (t, *J* = 5.2 Hz, 5.2H), 3.60 – 3.56 (m, 2.8H), 2.89 – 2.41 (m, 6H), 2.27 – 1.94 (m, 1.4H), 1.78 – 1.67 (m, 0.6H), 1.41 (t, *J* = 7.5 Hz, 1H), 1.03 (t, *J* = 7.6 Hz, 2H). ^13^C NMR (126 MHz, DMF-d_7_): δ (ppm) = 174.9, 174.8, 145.0, 149.8, 148.3, 145.1, 145.0, 144.9, 144.8, 141.5, 141.4, 141.4, 138.8, 138.7, 138.6, 137.5, 137.4, 137.3, 134.1, 134.0, 133.7, 133.6, 128.9, 128.8, 128.7, 128.3, 128.2, 128.1, 126.4, 126.3, 126.0, 125.9, 125.8, 124.9, 124.1, 124.0, 121.8, 121.7, 121.6, 73.5, 73.5, 73.3, 73.20, 73.10, 68.8, 68.7, 68.4, 68.3, 62.1, 62.0, 25.6, 23.9, 19.9, 12.8, 12.4. ^19^F NMR (471 MHz, CD_3_OD): δ (ppm) = –109.4 (m, 4F), –130.3 (m, 2F).

HRMS (ESI+, *m/z*): M^+^ calcd for C_51_H_52_F_6_NaO_14_S_2_^+^, 1089.2595; found, 1089.2632.

**Figure S1.** ^1^H NMR spectrum (400 MHz, CD_3_OD) of compound **A΄**.

**Figure S2.** ^13^C NMR spectrum (101 MHz, CD_3_OD) of compound **A΄**.

**Figure S3.** ^1^H NMR spectrum (400 MHz, CD_3_OD) of compound **A**.

**Figure S4.** ^13^C NMR spectrum (101 MHz, CD_3_OD) of compound **A**.

**Figure S5.** ^1^H NMR spectrum (400 MHz, CD_3_OD) of compound **B΄**.

**Figure S6.** ^13^C NMR spectrum (101 MHz, CD_3_OD) of compound **B΄**.

**Figure S7.** ^1^H NMR spectrum (400 MHz, CD_3_OD) of compound **B**.

**Figure S8.** ^13^C NMR spectrum (101 MHz, CD_3_OD) of compound **B**.

**Figure S9.** ^1^H NMR spectrum (400 MHz, CD_3_OD) of compound **C(Et)**.

**Figure S10.** ^13^C NMR spectrum (101 MHz, CD_3_OD) of compound **C(Et)**.

**Figure S11.** ^19^F NMR spectrum (376 MHz, CD_3_OD) of compound **C(Et)**.

**Figure S12.** ^1^H NMR spectrum (400 MHz, CD_3_OD) of compound **D(Et)**.

**Figure S13.** ^13^C NMR spectrum (101 MHz, CD_3_OD) of compound **D(Et)**.

**Figure S14.** ^19^F NMR spectrum (376 MHz, CD_3_OD) of compound **D(Et)**.

**Figure S15.** ^1^H NMR spectrum (400 MHz, DMF-d_7_) of compound **D(Et)΄**.

**Figure S16.** ^13^C NMR spectrum (101 MHz, DMF-d_7_) of compound **D(Et)΄**.

**Figure S17.** ^19^F NMR spectrum (376 MHz, CD_3_OD) of compound **D(Et)΄**.

**Figure S18.** ^1^H NMR spectrum (500 MHz, DMF-d_7_) of compound **HO-fDAE 2 (TPP)**.

**Figure S19.** ^13^C NMR spectrum (126 MHz, DMF-d_7_) of compound **HO-fDAE 2 (TPP)**.

**Figure S20.** ^19^F NMR spectrum (471 MHz, CD_3_OD) of compound **HO-fDAE 2 (TPP)**.

**Figure S21.** ^1^H NMR spectrum (400 MHz, CD_3_OD) of compound **C(iBu)**.

**Figure S22.** ^13^C NMR spectrum (101 MHz, CD_3_OD) of compound **C(iBu)**.

**Figure S23.** ^19^F NMR spectrum (376 MHz, CD_3_OD) of compound **C(iBu)**.

**Figure S24.** ^1^H NMR spectrum (500 MHz, CD_3_OD) of compound **D(iBu)**.

**Figure S25.** ^13^C NMR spectrum (126 MHz, CD_3_OD) of compound **D(iBu)**.

**Figure S26.** ^19^F NMR spectrum (471 MHz, CD_3_OD) of compound **D(iBu)**.

**Figure S27.** ^1^H NMR spectrum (500 MHz, DMF-d_7_) of compound **D(iBu)΄**.

**Figure S28.** ^13^C NMR spectrum (126 MHz, DMF-d_7_) of compound **D(iBu)΄**.

**Figure S29.** ^19^F NMR spectrum (471 MHz, DMF-d_7_) of compound **D(iBu)΄**.

**Figure S30.** ^1^H NMR spectrum (500 MHz, DMF-d_7_) of compound **HO-fDAE 3 (TPP)**.

**Figure S31.** ^13^C NMR spectrum (126 MHz, DMF-d_7_) of compound **HO-fDAE 3 (TPP)**.

**Figure S32.** ^19^F NMR spectrum (471 MHz, DMF-d_7_) of compound **HO-fDAE 3 (TPP)**.

**Figure S33.** ^1^H NMR spectrum (400 MHz, CD_3_OD) of compound **F**.

**Figure S34.** ^13^C NMR spectrum (101 MHz, CD_3_OD) of compound **F**.

**Figure S35.** ^19^F NMR spectrum (376 MHz, CD_3_OD) of compound **F**.

**Figure S36.** ^1^H NMR spectrum (400 MHz, CD_3_OD) of compound **F΄**.

**Figure S37.** ^13^C NMR spectrum (101 MHz, CD_3_OD) of compound **F΄**.

**Figure S38.** ^19^F NMR spectrum (376 MHz, CD_3_OD) of compound **F΄**.

**Figure S39.** ^1^H NMR spectrum (500 MHz, DMF-d_7_) of compound **HO-fDAE 1 (COOH)**.

**Figure S40.** ^13^C NMR spectrum (126 MHz, DMF-d_7_) of compound **HO-fDAE 1 (COOH)**.

**Figure S41.** ^19^F NMR spectrum (471 MHz, DMF-d_7_) of compound **HO-fDAE 1 (COOH)**.

**Geometry optimization, ΔG and HOMO/LUMO calculations**

Geometry optimizations were carried out starting from MMFF (Merck Molecular Force Field) optimized structures using DFT (density functional theory) at the B3LYP/6-31G(d,p) (Becke, 3-parameter, Lee-Yang-Parr) level, including Grimme’s D3 dispersion correction^[2]^ with Becke-Johnson, as implemented in Gaussian 16 which was used for all DFT calculations. The solvent effects of water were considered using the SMD (Solvation Model based on Density) model.^[3]^ Frequency calculations were performed at the same level to verify that all stationary points correspond to minima. For each compound in the open and closed form, the difference of the sum of electronic and thermal free energies (in Hartree, 1 H = 2625.499 kJ/mol) were calculated. All calculations were conducted on the scientific computing cluster of the *Gesellschaft für wissenschaftliche Datenverarbeitung mbH Göttingen* (GWDG) with 96 CPU cores (2 x Sapphire Rapids 8468) and 514 GB of RAM using the shared-memory multiprocessor parallel execution. Visualization has been carried out using the Avogadro software.

**Authors of Gaussian 16, Revision C.02:**

M. J. Frisch, G. W. Trucks, H. B. Schlegel, G. E. Scuseria, M. A. Robb, J. R. Cheeseman, G. Scalmani, V. Barone, G. A. Petersson, H. Nakatsuji, X. Li, M. Caricato, A. V. Marenich, J. Bloino, B. G. Janesko, R. Gomperts, B. Mennucci, H. P. Hratchian, J. V. Ortiz, A. F. Izmaylov, J. L. Sonnenberg, D. Williams-Young, F. Ding, F. Lipparini, F. Egidi, J. Goings, B. Peng, A. Petrone, T. Henderson, D. Ranasinghe, V. G. Zakrzewski, J. Gao, N. Rega, G. Zheng, W. Liang, M. Hada, M. Ehara, K. Toyota, R. Fukuda, J. Hasegawa, M. Ishida, T. Nakajima, Y. Honda, O. Kitao, H. Nakai, T. Vreven, K. Throssell, J. A. Montgomery, Jr., J. E. Peralta, F. Ogliaro, M. J. Bearpark, J. J. Heyd, E. N. Brothers, K. N. Kudin, V. N. Staroverov, T. A. Keith, R. Kobayashi, J. Normand, K. Raghavachari, A. P. Rendell, J. C. Burant, S. S. Iyengar, J. Tomasi, M. Cossi, J. M. Millam, M. Klene, C. Adamo, R. Cammi, J. W. Ochterski, R. L. Martin, K. Morokuma, O. Farkas, J. B. Foresman, and D. J. Fox, Gaussian, Inc., Wallingford CT, 2019.

**HO-fDAE1_open**

Zero-point correction=	0.972014 (Hartree/Particle)

Thermal correction to Energy=	1.044998

Thermal correction to Enthalpy=	1.045943

Thermal correction to Gibbs Free Energy=	0.850996

Sum of electronic and zero-point Energies=	-4422.545100

Sum of electronic and thermal Energies=	-4422.472116

Sum of electronic and thermal Enthalpies=	-4422.471172

Sum of electronic and thermal Free Energies=	-4422.666118

0 imaginary frequencies

Coordinates (Angstroms)

C	-0.0749644895	-3.1212726776	0.7102177802

C	0.5122521272	-2.9359956929	-0.4830508546

C	-0.0832521545	-5.2741761523	-0.3756502777

C	-0.2877904503	-4.5836369379	0.9999621723

F	0.5620905437	-6.4517436581	-0.2688719079

F	-1.2919686912	-5.4817545855	-0.9507539520

F	-1.5165305324	-4.8557755514	1.5114802292

F	0.6320609895	-5.0433611722	1.8951133319

F	0.2504908232	-4.1675592979	-2.4879992532

C	0.9059484701	-1.6261045581	-1.0304884793

C	-0.5011207862	-2.0371652278	1.6088665047

C	0.0358228426	-0.7501999917	-1.5723487529

S	0.9032744213	0.7945412158	-1.9251347456

C	2.4767421829	0.1617099158	-1.3714584423

C	2.3060805012	-1.1563071922	-0.9325499279

C	-1.9208308785	-1.6179019039	1.6283217471

C	-2.1377785900	-0.4556643344	2.3768697534

S	-0.5927581568	0.0653881633	3.1006447468

C	0.3402857708	-1.2865220713	2.3471264700

C	3.6782122262	0.8359097446	-1.3236787676

C	4.8046673627	0.1483555945	-0.8242998507

C	4.6505772727	-1.1839412864	-0.4047344360

C	3.4191076178	-1.8409430274	-0.4529607024

C	-3.0006561418	-2.1886717000	0.9601256909

C	-4.2439520235	-1.5555030421	1.0225964309

C	-4.4328891841	-0.3542087004	1.7251938847

C	-3.3487806283	0.1999979390	2.4352515888

C	0.7161205562	-4.2357393414	-1.2143081364

F	2.0277135883	-4.6044363539	-1.2816317595

C	1.7947557951	-1.4378215770	2.6428062672

C	-1.3870985499	-0.9028122802	-1.9901549714

C	-5.7100919579	0.3818110884	1.6132467744

O	0.8928503764	1.0774184118	-3.3778583920

O	0.4341814442	1.8693010519	-1.0213761800

O	-0.1791793408	1.3684717344	2.5338425945

O	-0.5909993464	-0.0623207431	4.5756297406

C	-5.6798646149	1.7661320178	1.4143134934

C	-6.8450805478	2.4674881753	1.0996977936

C	-8.0648605776	1.7917012149	1.0563150311

C	-8.1242402930	0.4153085899	1.3079273793

C	-6.9441927401	-0.2836782333	1.5620816144

C	6.1173076842	0.8276720801	-0.7439441117

C	7.3047963682	0.1168674064	-0.9624925947

C	8.5453206738	0.7539268810	-0.8738118981

C	8.5974953769	2.1158810783	-0.5713222676

C	7.4225485102	2.8452433151	-0.3578606550

C	6.1913270341	2.1964249769	-0.4392145476

C	-2.3428271592	0.2114915468	-1.5317463669

C	-3.7401546780	-0.0516946717	-2.0939722476

C	-4.8156595957	0.9346650545	-1.7081475998

O	-6.0075117215	0.6535126957	-1.7039319414

O	-4.3565139637	2.1559387664	-1.4091248050

C	2.6295498384	-0.1644401781	2.4448816932

C	-6.7337916704	3.8978027651	0.6565342024

O	-6.3629988410	3.8846561670	-0.7401283947

C	-6.1048573616	5.1949537310	-1.2502256902

C	-5.7895543717	5.0629286108	-2.7297412611

O	-5.5698863508	6.3825519389	-3.2261236856

C	-9.4487143293	-0.3024284714	1.2970928570

O	-10.1867570408	0.0954548772	0.1436534359

C	-11.4925631869	-0.4650219025	0.1236090658

C	-12.1826056097	0.0284471209	-1.1350481699

O	-13.4895794382	-0.5442822807	-1.1540247716

C	7.4843088351	4.3204535956	-0.0594175050

C	9.8170370124	-0.0020248343	-1.1624332810

O	8.5105272723	4.5691502462	0.8984042870

C	8.6885749238	5.9584366739	1.1392211820

C	9.8207697755	6.1130085956	2.1382513516

O	9.9812390068	7.5109697883	2.3770947755

O	9.6981497324	-1.3415464413	-0.6950519794

C	10.8048876678	-2.1437495980	-1.0836900190

C	10.5633016561	-3.5478440392	-0.5603425496

O	11.6731290339	-4.3450572083	-0.9722050815

H	3.7626241456	1.8570649932	-1.6780437827

H	5.5078673111	-1.7130431822	-0.0041821423

H	3.3329864031	-2.8638382318	-0.1069294452

H	-2.8768365756	-3.0879774086	0.3688043545

H	-5.0732669131	-1.9718687079	0.4622352035

H	-3.4672121332	1.1183289378	2.9993313360

H	1.8913263175	-1.7720643377	3.6840873368

H	2.1728539794	-2.2501004318	2.0169537012

H	-1.3996893796	-0.9428496532	-3.0883523995

H	-1.7399066992	-1.8763611755	-1.6380340116

H	-4.7299109800	2.2899564452	1.4088511569

H	-8.9714914218	2.3322553363	0.8036661888

H	-6.9837395817	-1.3570090681	1.7209330504

H	7.2674604290	-0.9361519314	-1.2159156233

H	9.5588953742	2.6137539834	-0.4921016778

H	5.2833603879	2.7546352440	-0.2337262633

H	-2.3693304229	0.2533694367	-0.4400620228

H	-1.9886442234	1.1841559419	-1.8810715988

H	-3.7012250106	-0.0454020981	-3.1919624446

H	-4.1010416752	-1.0419370641	-1.8045931994

H	-5.1207786918	2.7485837534	-1.1648161818

H	3.6891888379	-0.4066569077	2.5556131247

H	2.4768461450	0.2641892039	1.4528290374

H	2.3806928206	0.5976265554	3.1875485404

H	-7.6852251971	4.4308657586	0.7643770672

H	-5.9640606105	4.4316397174	1.2270181157

H	-6.9889935705	5.8299583216	-1.1078888535

H	-5.2613190565	5.6523593595	-0.7160266272

H	-4.9010529112	4.4322449320	-2.8645011850

H	-6.6314699654	4.5741805888	-3.2388119369

H	-5.3232976564	6.2988079523	-4.1576340926

H	-9.2945068519	-1.3904089237	1.2968600825

H	-10.0233485477	-0.0534109817	2.2030379669

H	-11.4439348178	-1.5640537732	0.1198417225

H	-12.0612262755	-0.1564805416	1.0134760670

H	-12.2248207745	1.1262882604	-1.1214733987

H	-11.5980672049	-0.2753473224	-2.0142252264

H	-13.9257106771	-0.2330224566	-1.9591749373

H	7.7017753730	4.8825523733	-0.9814712443

H	6.5145801177	4.6753672775	0.3165338703

H	10.0104019098	-0.0080876153	-2.2470585307

H	10.6727012121	0.4968872135	-0.6856454736

H	8.9355816681	6.4850576388	0.2053987507

H	7.7672366046	6.4040475404	1.5427727847

H	9.5705691821	5.5734135814	3.0620244522

H	10.7360955833	5.6678103815	1.7247930065

H	10.7109680269	7.6125771040	3.0034340734

H	10.9031179334	-2.1626129716	-2.1792437836

H	11.7411523827	-1.7420716002	-0.6688509606

H	10.4708042318	-3.5214538237	0.5340547563

H	9.6185944480	-3.9314430576	-0.9696775618

H	11.5184964156	-5.2421595871	-0.6460864398

LUMO+1 (-2.180 eV)

LUMO (-2.238eV)

HOMO (-6.161eV)

**HO-fDAE1_closed**

Zero-point correction=	0.972684 (Hartree/Particle)

Thermal correction to Energy=	1.045653

Thermal correction to Enthalpy=	1.046597

Thermal correction to Gibbs Free Energy=	0.847091

Sum of electronic and zero-point Energies=	-4422.544879

Sum of electronic and thermal Energies=	-4422.471909

Sum of electronic and thermal Enthalpies=	-4422.470965

Sum of electronic and thermal Free Energies=	-4422.670471

0 imaginary frequencies

Coordinates (Angstroms)

C	0.7796799936	-1.9685299913	-1.3126599910

C	-0.6769099961	-1.9475399921	-1.2680399911

C	0.0017900000	-3.9591399810	-2.4496999893

C	1.2468199956	-3.3202499862	-1.7674999938

F	0.0726399998	-3.7400599818	-3.7837999812

F	-0.0709299996	-5.2864299756	-2.2292499873

F	1.6537399912	-4.1193199799	-0.7371499949

F	2.2728899881	-3.2695399821	-2.6639699847

F	-1.8407099884	-3.9924899828	-0.9369899946

C	-1.3598499930	-0.9424299945	-0.6607699960

C	1.5147699916	-0.8633699935	-1.0174699950

C	-0.4872599973	0.0923199996	0.0638799998

S	-1.6447199910	1.5261599912	0.3847899978

C	-3.0961199853	0.5927699945	-0.0288499999

C	-2.7851699860	-0.6801199957	-0.5426899970

C	2.9393999852	-0.6750699941	-0.7997599985

C	3.2943099840	0.6186399971	-0.3719599980

S	1.8890799920	1.6872599912	-0.2029999988

C	0.6909499988	0.4284799980	-0.8922099979

C	-4.3826299808	1.0527699936	0.1842399990

C	-5.4580599881	0.2116299991	-0.1435199991

C	-5.1684499752	-1.0719599966	-0.6536099956

C	-3.8707199783	-1.5202699949	-0.8493299961

C	3.9851599789	-1.6107799937	-0.9055499944

C	5.2883699763	-1.2291399946	-0.6246699978

C	5.6240999737	0.0797799995	-0.2176599988

C	4.5873099781	1.0174699950	-0.0869099993

C	-1.2303299924	-3.2034699851	-1.8746699886

F	-2.1436899915	-2.9849899828	-2.8619699876

C	0.2735699987	0.9220299962	-2.3147199912

C	-0.0583299998	-0.4772499977	1.4525399902

C	7.0234899448	0.4567999979	0.0730699997

O	-1.6694399910	1.8844999897	1.8183399903

O	-1.4128799947	2.6335799885	-0.5685299953

O	2.0197899905	2.8511299882	-1.1047599927

O	1.6039999900	1.9885399913	1.2175499924

C	7.4694899682	1.7661199896	-0.1642099992

C	8.7875999606	2.1316199886	0.1110999992

C	9.6699799362	1.1769499930	0.6241399951

C	9.2463099739	-0.1326699994	0.8657799935

C	7.9243899585	-0.4858699975	0.5915299947

C	-6.8528299776	0.6618499991	0.0471099998

C	-7.8603099853	-0.2562199989	0.3779899982

C	-9.1781499386	0.1673699989	0.5579399952

C	-9.4894099616	1.5212799931	0.4092199981

C	-8.4990799522	2.4519599887	0.0811499996

C	-7.1862799885	2.0172999894	-0.1006099993

C	-1.1213899951	-1.2989299909	2.1877899902

C	-0.5809699989	-1.7406399906	3.5439899834

C	-1.5540799946	-2.5942899884	4.3107099774

C	1.3225199912	0.7193899982	-3.4102999817

O	-2.6445999874	-2.9665899833	3.9088299820

O	-1.0821899921	-2.9243699872	5.5331699723

C	-8.8687299705	3.8970899792	-0.1373399993

C	-10.2467399653	-0.8141199946	0.9657999946

C	9.2737699317	3.5247299809	-0.1964099990

C	10.1942999328	-1.1346199968	1.4731699901

O	-7.7768699581	4.7324599790	0.2276899987

C	-8.0099099696	6.0961099565	-0.0969099997

C	-6.7693699475	6.8781699457	0.2947299987

O	-7.0083699761	8.2446699658	-0.0401399998

O	-10.0294799653	-2.0560199916	0.3042799984

C	-10.9373799351	-3.0600199871	0.7370799953

C	-10.6120499368	-4.3319799801	-0.0245999999

O	-11.5334999653	-5.3311699612	0.4104399982

O	8.2473499838	4.4637199796	0.1066899994

O	9.9547899361	-2.4197699857	0.9085399938

C	8.5941899773	5.7840199719	-0.2882099984

C	7.4308899834	6.6905999621	0.0705599995

O	7.7866999540	8.0119999550	-0.3340699981

C	10.7365499348	-3.4314999849	1.5292299917

C	10.3962999439	-4.7500699752	0.8588399932

O	11.1815399706	-5.7596899914	1.4917899951

H	-4.5539799792	2.0327199910	0.6149099963

H	-5.9868999518	-1.7319599921	-0.9166399929

H	-3.7144899803	-2.5191399878	-1.2277999962

H	3.7941599797	-2.6267599844	-1.2159499934

H	6.0764699677	-1.9637099913	-0.7434699954

H	4.7893699767	2.0244999904	0.2593299988

H	-0.6356499963	0.3888499983	-2.5946199886

H	0.0088899999	1.9739099878	-2.2416399890

H	0.8170499959	-1.1103099927	1.2957899910

H	0.2638199986	0.3550399981	2.0746699903

H	6.7951899852	2.5029299882	-0.5851099957

H	10.6981099732	1.4565399931	0.8379499979

H	7.5945399401	-1.4960299904	0.8040999977

H	-7.6185599816	-1.3033599926	0.5173699974

H	-10.5143199553	1.8547499911	0.5489099989

H	-6.4262099582	2.7371599869	-0.3799399982

H	-2.0338999874	-0.7176599962	2.3424799872

H	-1.3956299924	-2.1819499905	1.6046999909

H	0.3455799982	-2.3173399898	3.4326099817

H	-0.3255699982	-0.8782599977	4.1704799804

H	0.9329199974	1.1348199940	-4.3438199762

H	2.2605799874	1.2333799952	-3.1881199830

H	1.5382499918	-0.3381099983	-3.5810399817

H	-1.7527799931	-3.4791899852	5.9714099823

H	-9.1207599295	4.0619899795	-1.1971499941

H	-9.7627399383	4.1534499783	0.4490299979

H	-10.2199599473	-0.9689299932	2.0562399917

H	-11.2417999194	-0.4170699981	0.7202099953

H	10.1819499422	3.7476699840	0.3813299981

H	9.5406199747	3.6035399827	-1.2623499920

H	11.2353699401	-0.8272299948	1.3005199938

H	10.0464299346	-1.1793799959	2.5639499889

H	-8.2026799660	6.2101799776	-1.1738799925

H	-8.8866999818	6.4806399616	0.4448499981

H	-6.5818699740	6.7543699433	1.3702699950

H	-5.9016299769	6.4775899429	-0.2469499985

H	-6.2026599461	8.7354699730	0.1727699994

H	-10.8389799624	-3.2311099849	1.8193799928

H	-11.9757899273	-2.7571599855	0.5370899951

H	-10.7025399273	-4.1444499811	-1.1032599923

H	-9.5733199511	-4.6255699771	0.1806799990

H	-11.3386999393	-6.1359399607	-0.0892299995

H	9.5054699607	6.1178599864	0.2295999988

H	8.7869499722	5.8266899644	-1.3704699922

H	6.5245899809	6.3423099701	-0.4439699981

H	7.2460099663	6.6375199426	1.1520899938

H	7.0591499797	8.5949299787	-0.0771899999

H	11.8095799171	-3.2171299856	1.4167399931

H	10.5151899227	-3.4868699817	2.6053999845

H	9.3212399320	-4.9484599764	0.9692099967

H	10.6182099299	-4.6824399743	-0.2149799988

H	10.9696399536	-6.5985899629	1.0599099965

LUMO+1 (-1.518 eV)

LUMO (-3.109 eV)

HOMO (-5.876 eV)

**Th-fDAE_open**

Zero-point correction=	1.281273 (Hartree/Particle)

Thermal correction to Energy=	1.382819

Thermal correction to Enthalpy=	1.383763

Thermal correction to Gibbs Free Energy=	1.111418

Sum of electronic and zero-point Energies=	-6547.563386

Sum of electronic and thermal Energies=	-6547.461840

Sum of electronic and thermal Enthalpies=	-6547.460896

Sum of electronic and thermal Free Energies=	-6547.733241

0 imaginary frequencies

Coordinates (Angstroms)

C	0.4911962026	2.6700222585	0.8894998433

C	-0.3171778663	2.7923934137	-0.1782023384

C	0.3860034673	5.0275505003	0.4027697751

C	0.7826403538	4.0174467367	1.5068267295

F	-0.1429645667	6.1609609409	0.9014895912

F	1.4850914424	5.3345705068	-0.3286416163

F	2.0771592240	4.1814635138	1.8810148816

F	0.0178648924	4.2177961012	2.6177312211

F	-0.4412983506	4.5214185043	-1.7990749765

C	-0.8403405771	1.7028021533	-1.0183866252

C	1.0430579885	1.4210061457	1.4340383039

C	-0.0619630553	0.9619618035	-1.8348865255

S	-1.0852156951	-0.2712167313	-2.6667051006

C	-2.5829053652	0.2812755962	-1.8696463506

C	-2.2769012995	1.3424528848	-1.0100812788

C	2.4885592497	1.1191734940	1.3175774761

C	2.8354024342	-0.1235464091	1.8579268588

S	1.3655560738	-0.9106698474	2.4964179969

C	0.2950641609	0.4645404724	2.0223054184

C	-3.8376213356	-0.2736575876	-1.9914219433

C	-4.8811179538	0.2619835685	-1.2053326030

C	-4.5848770277	1.3239923739	-0.3289599032

C	-3.3052507175	1.8683825008	-0.2298464429

C	3.4849564240	1.8606546509	0.6841723501

C	4.7767178365	1.3423261045	0.6096990560

C	5.1147291803	0.0925510754	1.1648995061

C	4.1034075736	-0.6593303906	1.8011974693

C	-0.6158980495	4.2412802872	-0.4833522258

F	-1.8910179183	4.6046408121	-0.1585796712

O	-1.0933044810	-0.0220852257	-4.1252278201

O	-0.7466518003	-1.6395015682	-2.2147315343

O	1.0623957376	-2.1239306416	1.7047197963

O	1.3874934589	-1.0506201689	3.9693321034

C	-6.2394772387	-0.2687332936	-1.2907340097

C	-7.4072187227	0.2977233129	-0.8476750050

C	-8.5693678091	-0.5002350044	-1.0824860297

C	-8.2878162708	-1.6892288796	-1.7344041928

S	-6.5796602888	-1.8229761277	-2.0370236411

C	-9.1842188074	-2.7979532722	-2.1963597505

C	-9.9026460311	0.0236638132	-0.6702929315

O	-10.0632244846	1.2426471317	-0.4800271853

N	-10.8931660152	-0.8852054750	-0.5299846745

C	-12.2643705794	-0.6193407600	-0.0816002410

C	-12.8686108868	0.5602897060	-0.8667452128

C	-12.2246105853	-0.3586692965	1.4329377953

C	-13.0502112223	-1.9021117428	-0.4078231396

O	-14.2881816658	0.4576914985	-0.8393106237

O	-13.5403530917	-0.0981092697	1.9013004083

O	-12.3273153087	-3.0099744814	0.1299237575

C	-14.9074535163	1.5558302376	-1.4905284736

C	-12.4525095344	-4.1965506100	-0.6463087259

C	-13.5810965617	0.0815522394	3.3091202941

C	-16.4190290027	1.3366077892	-1.4049762356

C	-11.5685262047	-5.2576297971	0.0116115467

C	-15.0310482509	0.3987255140	3.6815538956

C	12.8000352886	-4.9720007684	0.0797666679

C	11.1925744267	1.6249021194	-4.2545629632

C	16.6244741691	0.9734476584	2.0603375908

C	13.3305860293	-3.7897492030	-0.7328831068

C	12.0567786469	1.9324032062	-3.0300700812

C	15.1911834126	0.5400873501	2.3722836233

O	11.9045767512	0.8436320594	-2.1317732753

O	12.9085274644	-2.6077080332	-0.0665192716

O	14.5119907100	0.4052869060	1.1315855571

C	12.6816427364	1.0110319669	-0.9535363499

C	13.2196370424	-1.4198945772	-0.7864134667

C	13.1596954996	0.0004856794	1.3035122740

C	12.5358360001	-0.2426284670	-0.0800519791

N	11.1359645307	-0.6366555494	0.1047952052

O	10.2600202495	1.3658227527	0.7664217603

C	10.1337918528	0.1449289863	0.5604235931

C	8.5628360160	-1.8523040758	1.0420606693

C	8.8151522599	-0.5143100709	0.7902641834

C	6.4838382557	-0.4092953276	1.0741049634

S	6.8614750398	-2.1102529771	1.2992181861

C	7.6349638929	0.2915248359	0.8206443686

C	9.4853993276	-3.0304185761	1.1280098855

C	-1.8998617448	-0.7756302110	1.7183268526

C	-1.1573156029	0.4104861472	2.3556367561

C	2.1765214367	-0.2381192110	-1.8588147070

C	1.3891969756	1.0459061049	-2.1681082388

C	-17.1816509372	2.4464089833	-2.0889243521

O	-17.7746448531	2.3223601564	-3.1453985651

O	-17.1756310702	3.6587929732	-1.4858950403

C	-11.4802394964	-6.5026443670	-0.8387302168

O	-12.0510695128	-7.5493744537	-0.5921143492

O	-10.7271227487	-6.4172804103	-1.9607244740

C	-15.1875599246	0.5736101071	5.1729858176

O	-15.3322000897	1.6463670709	5.7307985033

O	-15.1317315174	-0.5513149399	5.9244031180

C	13.2294657682	-6.2900964254	-0.5195068875

O	14.0772978277	-7.0206802255	-0.0401137971

O	12.6374319386	-6.6526388530	-1.6820696941

C	11.3188257367	2.6943028985	-5.3127269713

O	11.8927941444	2.5454302735	-6.3764423763

O	10.7705714255	3.9000132600	-5.0326388408

C	17.4481856435	1.1068739869	3.3192311084

O	17.8095858949	2.1667425594	3.7973521778

O	17.7773006876	-0.0417703793	3.9564252755

H	-4.0126593446	-1.0923161684	-2.6812569393

H	-5.3700293486	1.7244803369	0.3014332504

H	-3.1164210222	2.6794433167	0.4617912026

H	3.2604481302	2.8201006931	0.2344918870

H	5.5380476632	1.9129743803	0.0911065635

H	4.3129666483	-1.6274231884	2.2432595570

H	-7.4631174575	1.2694074961	-0.3748433740

H	-9.4594412805	-3.4556043560	-1.3624494385

H	-10.1037007201	-2.3999655710	-2.6321647993

H	-8.6909363242	-3.4160884713	-2.9498239030

H	-10.6512351915	-1.8670477889	-0.4901459388

H	-12.5107178102	0.5202934697	-1.9045981666

H	-12.5508785502	1.5132018014	-0.4322577316

H	-11.8045298203	-1.2376877146	1.9397384596

H	-11.5696782764	0.4993681788	1.6325021836

H	-14.0520040038	-1.8554888442	0.0241301914

H	-13.1376131993	-1.9997143513	-1.4960987464

H	-14.6218166770	2.4979923785	-1.0008264510

H	-14.5868906638	1.6111975991	-2.5408416986

H	-13.4972453109	-4.5317998075	-0.6768835584

H	-12.1247054209	-4.0045475395	-1.6767934904

H	-13.2385675633	-0.8297558688	3.8204132231

H	-12.9204133137	0.9064791555	3.6107444870

H	-16.6847294611	0.3970255200	-1.8912955088

H	-16.7132997915	1.2897186403	-0.3510290603

H	-10.5632840874	-4.8436581025	0.1445975997

H	-11.9704042792	-5.5352435531	0.9868015934

H	-15.3449203985	1.3233540962	3.1950327254

H	-15.6734752557	-0.4205141410	3.3418788504

H	11.7061956772	-4.9235490969	0.1107291645

H	13.1870899453	-4.9228633943	1.0984964767

H	10.1454147539	1.5447644119	-3.9435963795

H	11.5056702713	0.6777532453	-4.6960252868

H	16.6205004187	1.9393092916	1.5536005177

H	17.0868626519	0.2274402091	1.4050585915

H	14.4269671730	-3.8234055032	-0.7940555052

H	12.9321812094	-3.8097630714	-1.7564028779

H	13.1103810522	2.0438510114	-3.3235218571

H	11.7393396862	2.8693141523	-2.5506169881

H	15.1818612152	-0.4168742035	2.9134812400

H	14.6929798988	1.2893307534	3.0032647473

H	13.7412032008	1.1389691296	-1.2088460063

H	12.3509185173	1.8961382529	-0.4017116504

H	14.3018056505	-1.2492700702	-0.8105502075

H	12.8498311285	-1.4952156502	-1.8161719618

H	13.1072709190	-0.9272349673	1.8872265594

H	12.5911457078	0.7760935215	1.8312247356

H	10.9735187696	-1.6272294301	-0.0049458247

H	7.6653161746	1.3629102036	0.6717680394

H	10.3846449746	-2.7857807994	1.6994128103

H	8.9997510193	-3.8772577616	1.6174374663

H	9.7954189063	-3.3665710792	0.1310878421

H	-2.9763823334	-0.6476152486	1.8565567873

H	-1.6968172332	-0.8421122783	0.6478213472

H	-1.6082172678	-1.7233741977	2.1768514634

H	-1.2589748574	0.3595768089	3.4469331524

H	-1.6026498579	1.3567010870	2.0388212455

H	3.2466432198	-0.0433131660	-1.9645939448

H	1.9893631847	-0.5824182511	-0.8400701041

H	1.9089172210	-1.0466740998	-2.5430183420

H	1.4833746328	1.2820971656	-3.2355081883

H	1.8060532946	1.8928501150	-1.6183317599

H	-16.7017093818	3.6309055936	-0.6379546583

H	-10.2992340269	-5.5472286231	-2.0266543343

H	-15.0450764741	-1.3388076106	5.3616705183

H	11.9558556102	-6.0106435510	-1.9413262134

H	10.3125370657	3.8862829212	-4.1760130150

H	17.4644919217	-0.8174885886	3.4619360301

LUMO+1 (-2.287 eV)

LUMO (-2.379 eV)

HOMO (-5.874 eV)

**Th-fDAE_closed**

Zero-point correction=	1.282856 (Hartree/Particle)

Thermal correction to Energy=	1.384311

Thermal correction to Enthalpy=	1.385255

Thermal correction to Gibbs Free Energy=	1.121702

Sum of electronic and zero-point Energies=	-6547.525002

Sum of electronic and thermal Energies=	-6547.423548

Sum of electronic and thermal Enthalpies=	-6547.422603

Sum of electronic and thermal Free Energies=	-6547.686156

0 imaginary frequencies

Coordinates (Angstroms)

C	0.6712510491	-2.4543206377	0.4653975760

C	-0.7830605469	-2.3996800005	0.4133960906

C	-0.1306411985	-4.6946660052	0.8338415666

C	1.1204344968	-3.8831575159	0.3953725503

F	-0.2083437054	-5.8896950729	0.2170441244

F	-0.0674403347	-4.8932303015	2.1733539883

F	2.1606204236	-4.1693780800	1.2284364535

F	1.4935363698	-4.2735512755	-0.8595562028

F	-2.2981282219	-3.9225790960	1.4857946078

C	-1.4490611686	-1.2265556095	0.2400449980

C	1.4174945363	-1.3310777233	0.6466050599

C	-0.5566998419	0.0041912075	0.0190078434

S	-1.7015031926	1.4553475928	0.2960844135

C	-3.1648759906	0.4518856387	0.2251620874

C	-2.8676117627	-0.9230857894	0.1990924453

C	2.8424431581	-1.0850931483	0.5387348895

C	3.2138598504	0.2615810594	0.7127330759

S	1.8150049832	1.3184388601	0.9877265105

C	0.5976578297	-0.0977011900	1.0560836378

C	-4.4418167793	0.9739473551	0.1575178782

C	-5.5287270004	0.0856461998	0.0773842042

C	-5.2530933725	-1.3027054957	0.0560651520

C	-3.9639161969	-1.8031050155	0.1067154565

C	3.8806320071	-1.9890100118	0.2345474262

C	5.1900754210	-1.5468854230	0.1697480604

C	5.5434294483	-0.1931572340	0.3829592601

C	4.5134848097	0.7258787056	0.6509663109

C	-1.3506651065	-3.7856398192	0.5160096453

F	-1.9227145007	-4.1984327769	-0.6571643572

O	-1.5085125981	2.0455130564	1.6390261554

O	-1.6768392060	2.3913661133	-0.8467043405

O	1.9328564147	1.9878630149	2.2998034170

O	1.5628242987	2.1945377722	-0.1778486057

C	-6.9012376269	0.5671999401	0.0054728234

C	-8.0422538627	-0.1441228261	-0.2751761484

C	-9.2362407371	0.6369111314	-0.2654246597

C	-9.0036674598	1.9744277189	0.0138474936

S	-7.3080641433	2.2560520350	0.2743418153

C	-9.9430064297	3.1374525459	0.1124417100

C	-10.5324625313	-0.0303671973	-0.5889774397

O	-10.5304911875	-1.1236663620	-1.1833872379

N	-11.6597637639	0.5911999363	-0.1851570954

C	-13.0368370092	0.1742721573	-0.4653613543

C	-13.9319698381	1.0490083692	0.4211984629

C	-13.3245912982	0.4215991215	-1.9545102667

C	-13.2859166994	-1.3011361043	-0.1207587189

O	-13.5534759986	2.4067319536	0.2283461451

O	-14.6482619376	-0.0062127424	-2.2435539065

O	-12.7514679865	-1.5719524309	1.1667306127

C	-14.1457788090	3.2923542615	1.1722965144

C	-12.8981227822	-2.9393257575	1.5269946360

C	-14.9896098654	0.1989109857	-3.6101166606

C	-13.5460929182	4.6710655007	0.9187805094

C	-12.2399016370	-3.1128506636	2.8914098725

C	-16.4195329911	-0.2939961183	-3.7941505763

C	16.5067928839	-3.1641367512	-1.9234954869

C	12.3004293494	4.1426012000	-2.3299255014

C	13.5897974521	3.0389412449	2.8077881894

C	15.0200681414	-3.1709146328	-1.5900212555

C	12.6904146953	2.7350521841	-2.7692578337

C	14.2399996047	1.7747064349	2.2589286793

O	12.8083813657	1.9512922660	-1.5892571289

O	14.6592996746	-1.8334527833	-1.2639014178

O	13.4769809989	1.3831122791	1.1237206397

C	13.1280773201	0.5956976356	-1.8677799215

C	13.2822188091	-1.7207313333	-0.9263782135

C	13.9616846729	0.1919486387	0.5180233984

C	12.9880802617	-0.2491362513	-0.5916857316

N	11.6371103755	-0.1210315616	-0.0273794532

O	10.4134041683	-1.0050790090	-1.7485322877

C	10.4843861469	-0.3381649878	-0.7016691076

C	9.1264428694	1.4758385831	0.5067883667

C	9.2465426531	0.2396212466	-0.1036779364

C	6.9363503339	0.2245408593	0.3123389699

S	7.4709770091	1.7726901268	0.9533549850

C	8.0052418329	-0.4529774826	-0.2223270592

C	10.1593598963	2.5215605502	0.7910561711

C	1.1441858961	-0.9314514460	3.4721189553

C	0.1432383043	-0.2366896531	2.5463942651

C	-1.1537439002	-0.3446439928	-2.5069366640

C	-0.0970711246	0.0514464075	-1.4738315939

C	-14.1068999478	5.7485845727	1.8082242229

O	-14.3974207752	6.8746784874	1.4413921050

O	-14.2368188792	5.3490426039	3.0906229666

C	-12.3347694576	-4.5119967211	3.4391499505

O	-12.5871173992	-4.7907426571	4.5995025332

O	-12.0808373183	-5.4546348357	2.5073538461

C	-16.9254624392	-0.2016668930	-5.2093856106

O	-17.6663248186	-1.0150076223	-5.7359671359

O	-16.4973747997	0.9068634546	-5.8481577841

C	17.0497236725	-4.5051464745	-2.3407603200

O	17.8966339582	-4.6741552183	-3.2018636306

O	16.5211804185	-5.5266497153	-1.6352528203

C	12.1680266189	5.1155393348	-3.4710950609

O	12.5948172451	6.2580212485	-3.4692849151

O	11.4899343933	4.5974270208	-4.5168133185

C	14.3144791271	3.6461019279	3.9794055049

O	14.4350524186	4.8437257260	4.1762581881

O	14.8047149255	2.7201609099	4.8296542849

H	-4.5872627808	2.0486966500	0.1537204983

H	-6.0762095067	-2.0043495944	-0.0057659772

H	-3.8175700545	-2.8718323796	0.0557248366

H	3.6720247423	-3.0336834989	0.0580023227

H	5.9697360890	-2.2694719954	-0.0394863982

H	4.7204411633	1.7826294454	0.7841032959

H	-8.0535882564	-1.2024885983	-0.4988619108

H	-10.5203606719	3.1008192271	1.0441918141

H	-9.4011069547	4.0853584450	0.1049416666

H	-10.6462271114	3.1475077913	-0.7248947006

H	-11.5926022860	1.4944585564	0.2612000396

H	-13.7955909230	0.7643069882	1.4718889367

H	-14.9813118376	0.8921033475	0.1461603245

H	-12.6002244307	-0.1367751482	-2.5611661040

H	-13.2076537716	1.4909803857	-2.1731171921

H	-14.3673811843	-1.4885640197	-0.1354068700

H	-12.8165874754	-1.9455423482	-0.8701241680

H	-15.2367206989	3.3101362244	1.0421029136

H	-13.9303843702	2.9535299427	2.1927688069

H	-13.9626098864	-3.2118911312	1.5713105125

H	-12.4166125339	-3.5803901679	0.7777725267

H	-14.9053894606	1.2629283985	-3.8644565680

H	-14.2999277064	-0.3590143301	-4.2594491051

H	-12.4638012764	4.6285430005	1.0942988155

H	-13.7069670758	4.9741017766	-0.1171824965

H	-11.1749382204	-2.8631179052	2.8082334126

H	-12.6879238582	-2.4365442047	3.6214473712

H	-16.5098353354	-1.3343245112	-3.4759014823

H	-17.0924418730	0.3048904705	-3.1676803493

H	17.0732711057	-2.8437159033	-1.0401293525

H	16.7160235195	-2.4553275772	-2.7268696069

H	13.0353816131	4.5424112182	-1.6293437471

H	11.3313249024	4.1017716487	-1.8173459309

H	12.5696757184	2.8068323366	3.1388787632

H	13.5187060397	3.7996267772	2.0280762388

H	14.4298603435	-3.5226529339	-2.4482124192

H	14.8117311274	-3.8357177423	-0.7422882051

H	11.9277964121	2.3075093220	-3.4323045565

H	13.6440797622	2.7499293981	-3.3163597928

H	15.2810344746	1.9681860473	1.9651139249

H	14.2423821192	0.9734974751	3.0084562667

H	12.4508647987	0.2019646827	-2.6349034660

H	14.1558892687	0.5091945307	-2.2419241942

H	12.6550876186	-2.0553303679	-1.7591402745

H	13.0489650100	-2.3386069339	-0.0484138672

H	14.9578450510	0.3511825772	0.0917987416

H	14.0337753307	-0.6025556241	1.2742955324

H	11.5986557028	0.4811158181	0.7849063594

H	7.9249508864	-1.4196668723	-0.7027888995

H	10.9357246159	2.5131121144	0.0236818301

H	9.7139594307	3.5187776634	0.8199130880

H	10.6403175778	2.3479014553	1.7611477823

H	0.7474287171	-0.9085911864	4.4909239057

H	1.2967794095	-1.9780790099	3.1974934100

H	2.1157916580	-0.4315530242	3.4868435916

H	-0.0840679859	0.7579147657	2.9216734536

H	-0.7924231047	-0.7960937477	2.5596535726

H	-0.7081973869	-0.2640427695	-3.5022913327

H	-1.4880134425	-1.3767545589	-2.3752502701

H	-2.0282537089	0.3093092007	-2.4847709495

H	0.2713718998	1.0538370795	-1.6792842217

H	0.7547259491	-0.6232490512	-1.5720342576

H	-14.5908722989	6.0995844282	3.6023070263

H	-12.1471445454	-6.3267148532	2.9381151905

H	-16.8783542903	0.8997615260	-6.7455528985

H	16.9324497479	-6.3520712258	-1.9514676036

H	11.4314585125	5.2839980094	-5.2064997797

H	15.2423276489	3.1909063392	5.5628414675

LUMO+1 (-1.757 eV)

LUMO (-3.146 eV)

HOMO (-5.661 eV)

1. Uno, K.; Bossi, M. L.; Konen, T., Belov, V. N.; Irie, M.; Hell, S. W. *Adv. Opt. Mat.* **2019**, *7*, 1801746–1801746.

   .
2. S. Grimme, S. Ehrlich and L. Goerigk, “Effect of the damping function in dispersion corrected density functional theory,” *J. Comp. Chem*. **2011**, *32*, 1456-1465.
3. A. V. Marenich, C. J. Cramer, and D. G. Truhlar, “Universal solvation model based on solute electron density and a continuum model of the solvent defined by the bulk dielectric constant and atomic surface tensions,” *J. Phys. Chem. B* **2009**, *113***,** 6378-6396.
